# Supplementary material for: Collective search in ants: Movement determines footprints, and footprints influence movement
Source: PLoS One. 2024 Apr 23;19(4):e0299432. doi: 10.1371/journal.pone.0299432 (PMC11037541; doi:10.1371/journal.pone.0299432)
Supplement: S1 File — Example track color coded by straightness values, correlation between speed and straightness per point, mean track distance to the nest, heatmaps separated by colony, statistics to Figs 5–7. (PDF) [file pone.0299432.s001.pdf]

S1: Main analyses supplement

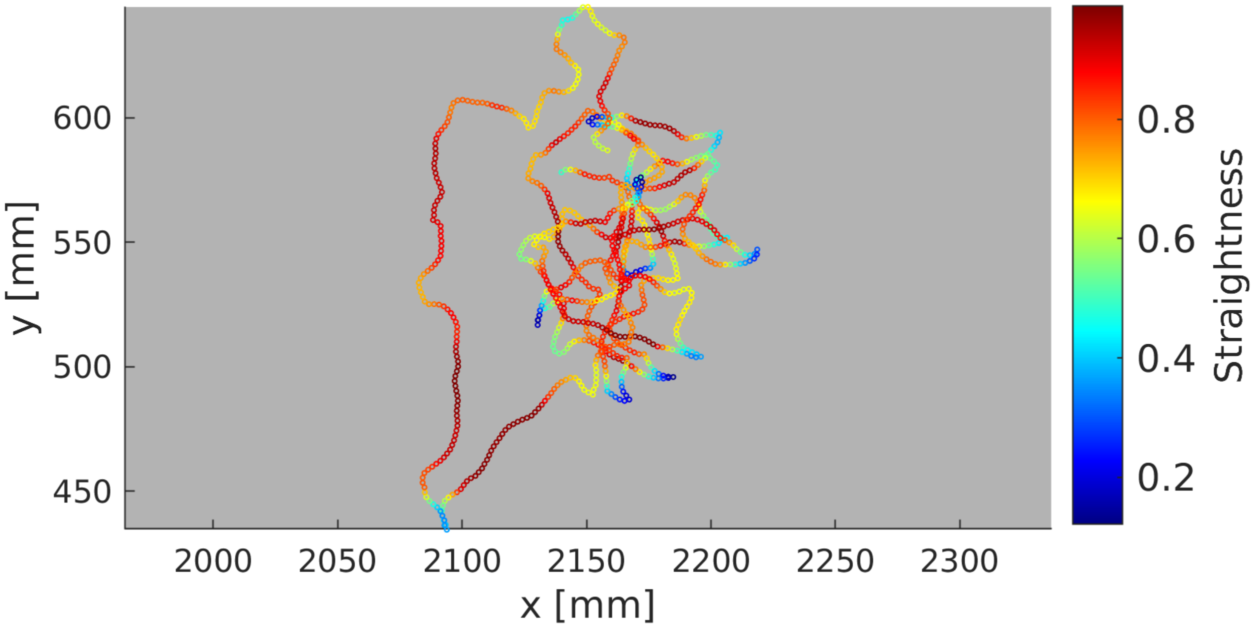

Fig S1.1 Example track *illustrating local straightness values (see main text Methods) as colors.*

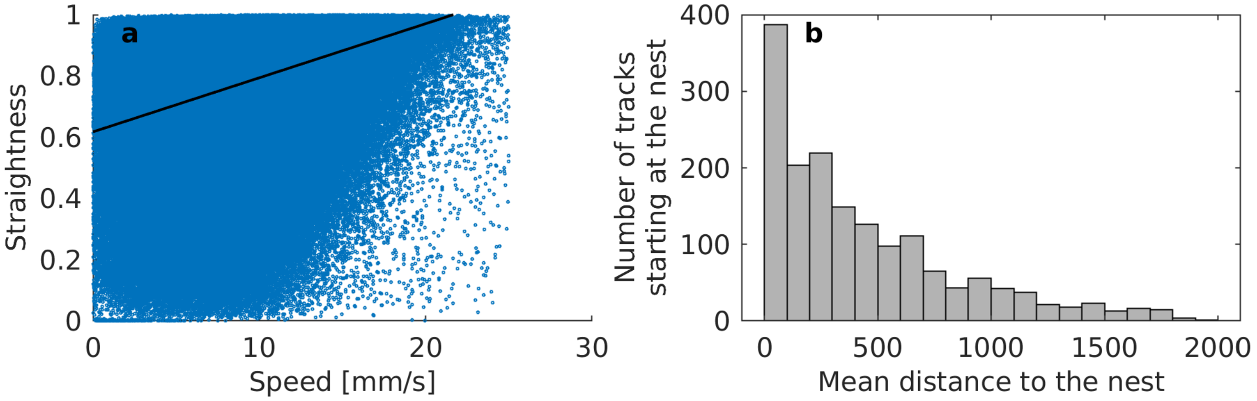

Fig S1.2 *a) The overall correlation of straightness vs speed is significantly positive (Estimate = 1.77e-2, SE = 4.44e-5, t = 396.9, DF = 1.78e6, p < 0.001), Lower = 1.76e-2, Upper = 1.77e-2. b) There are disproportionately many tracks which have their mean distance to the nest at 3-7cm.*

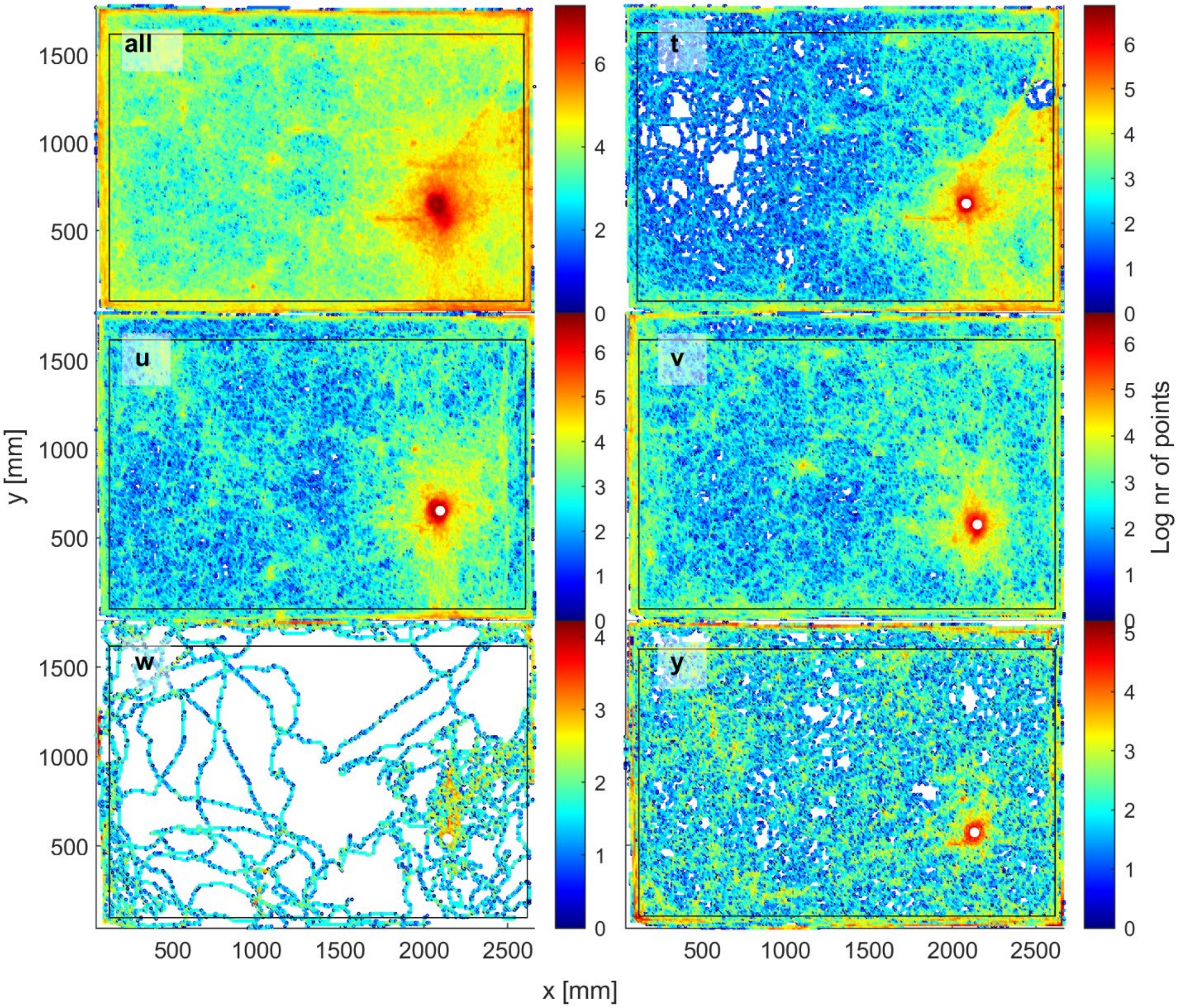

Fig S1.3: *Main text fig. 3a split up by colonies. Heatmaps of footprint concentration (= number of points), binned into pixels of size 10mm. all: All colonies combined, a copy of fig 2 of the main manuscript. t-y: individual colonies. White circles indicate nest locations. Note that the faint red (high concentration of points) horizontal lines to the top and left of the nest location likely correspond to the locations of adhesive tape beneath the paper overlay(s), and the circle of somewhat lower concentration in the center of the arena (i.e. faint bluish circle to the left of the nest location) may correspond to a drain in the floor of the room (again, under several layers of paper overlay).*

Table S1.4. **Stats to fig. 5a:** Lower straightness on higher footprint concentration irrespective of distance to the nest. Rows are nest distance bins, and pCorr stands for Benjamini-Hochberg corrected p-values.

**Nest dist.**	**slope**	**SE**	**tStat**	**DF**	**pValue**	**Lower**	**Upper**	**pCorr**
ND bin01	-9.95E-04	5.33E-05	-18.66	7.49E+04	<0.001	-1.10E-03	-8.90E-04	<0.001
ND bin02	-7.43E-04	6.22E-05	-11.95	8.89E+04	<0.001	-8.65E-04	-6.21E-04	<0.001
ND bin03	-2.96E-03	1.22E-04	-24.30	8.96E+04	<0.001	-3.20E-03	-2.72E-03	<0.001
ND bin04	-4.60E-03	1.89E-04	-24.30	8.98E+04	<0.001	-4.97E-03	-4.23E-03	<0.001
ND bin05	-6.20E-03	2.31E-04	-26.82	8.94E+04	<0.001	-6.65E-03	-5.74E-03	<0.001

ND bin06	-5.96E-03	2.39E-04	-24.96	8.95E+04	<0.001	-6.43E-03	-5.49E-03	<0.001
ND bin07	-7.65E-03	2.75E-04	-27.85	8.93E+04	<0.001	-8.18E-03	-7.11E-03	<0.001
ND bin08	-8.06E-03	2.51E-04	-32.12	8.95E+04	<0.001	-8.55E-03	-7.56E-03	<0.001
ND bin09	-7.12E-03	2.78E-04	-25.65	8.97E+04	<0.001	-7.66E-03	-6.58E-03	<0.001
ND bin10	-8.95E-03	2.80E-04	-31.94	8.96E+04	<0.001	-9.49E-03	-8.40E-03	<0.001
ND bin11	-9.59E-03	1.56E-04	-61.63	8.96E+04	<0.001	-9.90E-03	-9.29E-03	<0.001
ND bin12	-9.41E-03	2.29E-04	-41.14	8.93E+04	<0.001	-9.86E-03	-8.96E-03	<0.001
ND bin13	-1.01E-02	3.15E-04	-31.96	8.94E+04	<0.001	-1.07E-02	-9.46E-03	<0.001
ND bin14	-4.11E-03	5.22E-05	-78.75	8.98E+04	<0.001	-4.21E-03	-4.01E-03	<0.001
ND bin15	-1.26E-02	3.34E-04	-37.76	8.97E+04	<0.001	-1.33E-02	-1.20E-02	<0.001
ND bin16	-1.43E-02	3.26E-04	-44.03	9.01E+04	<0.001	-1.50E-02	-1.37E-02	<0.001
ND bin17	-1.29E-02	4.07E-04	-31.79	9.00E+04	<0.001	-1.37E-02	-1.21E-02	<0.001
ND bin18	-9.06E-03	4.17E-04	-21.72	9.01E+04	<0.001	-9.87E-03	-8.24E-03	<0.001
ND bin19	-1.22E-02	4.03E-04	-30.32	9.01E+04	<0.001	-1.30E-02	-1.14E-02	<0.001
ND bin20	-5.43E-03	3.33E-04	-16.31	9.01E+04	<0.001	-6.08E-03	-4.78E-03	<0.001

Table S1.5. **Stats to fig. 5b:** Mostly lower speed on higher footprint concentration irrespective of distance to the nest. pCorr stands for Benjamini-Hochberg corrected p-values.

Nest dist.	slope	SE	tStat	DF	pValue	Lower	Upper	pCorr
ND bin 01	-5.02E-02	8.29E-04	-60.60	89505	<0.001	-5.18E-02	-4.86E-02	<0.001
ND bin 02	-3.97E-02	1.15E-03	-34.36	90181	<0.001	-4.19E-02	-3.74E-02	<0.001
ND bin 03	-6.94E-02	2.29E-03	-30.38	90206	<0.001	-7.39E-02	-6.50E-02	<0.001
ND bin 04	-9.93E-02	3.68E-03	-26.98	90216	<0.001	-1.07E-01	-9.21E-02	<0.001
ND bin 05	-7.09E-02	4.34E-03	-16.34	90210	<0.001	-7.94E-02	-6.24E-02	<0.001
ND bin 06	-5.07E-02	4.54E-03	-11.17	90222	<0.001	-5.96E-02	-4.18E-02	<0.001
ND bin 07	-3.89E-02	5.07E-03	-7.67	90219	<0.001	-4.88E-02	-2.89E-02	<0.001
ND bin 08	-1.05E-01	4.79E-03	-21.90	90221	<0.001	-1.14E-01	-9.54E-02	<0.001
ND bin 09	-3.38E-02	5.36E-03	-6.30	90223	<0.001	-4.43E-02	-2.33E-02	<0.001
ND bin 10	2.31E-03	5.18E-03	0.45	90222	<0.001	-7.83E-03	1.25E-02	0.655
ND bin 11	3.13E-02	2.85E-03	10.98	90229	<0.001	2.57E-02	3.68E-02	<0.001
ND bin 12	1.72E-02	4.05E-03	4.24	90206	<0.001	9.24E-03	2.51E-02	<0.001
ND bin 13	-5.46E-02	5.31E-03	-10.29	90214	<0.001	-6.50E-02	-4.42E-02	<0.001
ND bin 14	-3.80E-02	8.43E-04	-45.07	90228	<0.001	-3.96E-02	-3.63E-02	<0.001
ND bin 15	-7.18E-02	5.46E-03	-13.15	90230	<0.001	-8.25E-02	-6.11E-02	<0.001
ND bin 16	-1.21E-01	5.33E-03	-22.66	90243	<0.001	-1.31E-01	-1.10E-01	<0.001
ND bin 17	-1.71E-02	7.15E-03	-2.40	90242	0.002	-3.12E-02	-3.13E-03	0.017
ND bin 18	-7.92E-02	7.74E-03	-10.22	90242	<0.001	-9.43E-02	-6.40E-02	<0.001
ND bin 19	-4.67E-02	7.27E-03	-6.42	90246	<0.001	-6.10E-02	-3.25E-02	<0.001
ND bin 20	-2.20E-02	6.26E-03	-3.50	90246	<0.001	-3.42E-02	-9.67E-03	<0.001

Table S1.6. **Stats to fig. 6a:** Lower maximum number of footprints in pixel with higher straightness of the first point of the pixels, irrespective of distance to the nest.

Nest dist.	slope	SE	tStat	DF	pValue	Lower	Upper	pCorr

ND bin01	-1.57	2.56E-01	-6.13	71143	>0.001	-2.07	-1.07	>0.001
ND bin02	-0.86	2.05E-01	-4.22	87468	>0.001	-1.27	-0.46	>0.001
ND bin03	-3.38	1.23E-01	-27.44	89801	>0.001	-3.63	-3.14	>0.001
ND bin04	-2.10	7.23E-02	-29.02	89834	>0.001	-2.24	-1.96	>0.001
ND bin05	-1.35	6.16E-02	-22.00	89535	>0.001	-1.47	-1.23	>0.001
ND bin06	-0.95	5.98E-02	-15.84	89536	>0.001	-1.06	-0.83	>0.001
ND bin07	-1.30	5.22E-02	-24.98	89347	>0.001	-1.41	-1.20	>0.001
ND bin08	-2.42	6.00E-02	-40.41	89583	>0.001	-2.54	-2.31	>0.001
ND bin09	-0.81	5.15E-02	-15.70	89834	>0.001	-0.91	-0.71	>0.001
ND bin10	-1.38	5.25E-02	-26.33	89715	>0.001	-1.49	-1.28	>0.001
ND bin11	-5.58	1.20E-01	-46.39	89660	>0.001	-5.81	-5.34	>0.001
ND bin12	-1.93	6.87E-02	-28.11	89404	>0.001	-2.06	-1.80	>0.001
ND bin13	-1.61	4.90E-02	-32.88	89496	>0.001	-1.71	-1.52	>0.001
ND bin14	-1.40	3.73E-02	-37.43	89510	>0.001	-1.47	-1.32	>0.001
ND bin15	-2.10	4.26E-02	-49.30	89686	>0.001	-2.18	-2.02	>0.001
ND bin16	-2.82	4.39E-02	-64.10	90118	>0.001	-2.90	-2.73	>0.001
ND bin17	-1.57	3.47E-02	-45.13	89982	>0.001	-1.63	-1.50	>0.001
ND bin18	-1.03	3.28E-02	-31.39	90060	>0.001	-1.09	-0.97	>0.001
ND bin19	-1.39	3.42E-02	-40.66	90101	>0.001	-1.46	-1.32	>0.001
ND bin20	-0.88	4.14E-02	-21.33	90151	>0.001	-0.96	-0.80	>0.001

Table S1.7. **Stats to fig. 6b:** Lower maximum number of footprints in pixel with higher speed of the first point of the pixels, irrespective of distance to the nest.

**Nest dist.**	**slope**	**SE**	**tStat**	**DF**	**pValue**	**Lower**	**Upper**	**pCorr**
ND bin 01	-4.12E-02	1.29E-02	-3.20	88889	0.002	-6.64E-02	-1.59E-02	0.001
ND bin 02	1.48E-01	1.08E-02	13.80	90042	>0.001	1.27E-01	1.69E-01	>0.001
ND bin 03	-5.82E-02	6.96E-03	-8.36	90220	>0.001	-7.18E-02	-4.45E-02	>0.001
ND bin 04	-1.03E-01	3.75E-03	-27.39	90224	>0.001	-1.10E-01	-9.54E-02	>0.001
ND bin 05	-5.20E-02	3.22E-03	-16.15	90226	>0.001	-5.84E-02	-4.57E-02	>0.001
ND bin 06	-3.10E-02	3.06E-03	-10.12	90222	>0.001	-3.70E-02	-2.50E-02	>0.001
ND bin 07	-2.36E-02	2.78E-03	-8.47	90216	>0.001	-2.90E-02	-1.81E-02	>0.001
ND bin 08	-9.07E-02	3.12E-03	-29.09	90225	>0.001	-9.68E-02	-8.46E-02	>0.001
ND bin 09	-3.27E-02	2.64E-03	-12.41	90231	>0.001	-3.79E-02	-2.76E-02	>0.001
ND bin 10	-3.29E-02	2.80E-03	-11.75	90228	>0.001	-3.84E-02	-2.74E-02	>0.001
ND bin 11	-1.67E-01	6.63E-03	-25.28	90232	>0.001	-1.80E-01	-1.55E-01	>0.001
ND bin 12	-5.58E-02	3.90E-03	-14.31	90200	>0.001	-6.35E-02	-4.82E-02	>0.001
ND bin 13	-4.93E-02	2.94E-03	-16.75	90213	>0.001	-5.51E-02	-4.35E-02	>0.001
ND bin 14	-4.81E-01	1.65E-02	-29.13	90069	>0.001	-5.14E-01	-4.49E-01	>0.001
ND bin 15	-7.25E-02	2.65E-03	-27.37	90221	>0.001	-7.77E-02	-6.73E-02	>0.001
ND bin 16	-1.09E-01	2.80E-03	-38.91	90242	>0.001	-1.14E-01	-1.03E-01	>0.001
ND bin 17	-3.50E-02	2.00E-03	-17.50	90240	>0.001	-3.89E-02	-3.11E-02	>0.001
ND bin 18	-2.69E-02	1.78E-03	-15.11	90240	>0.001	-3.04E-02	-2.34E-02	>0.001
ND bin 19	-3.14E-02	1.91E-03	-16.44	90242	>0.001	-3.52E-02	-2.77E-02	>0.001
ND bin 20	-2.17E-02	2.21E-03	-9.80	90242	>0.001	-2.60E-02	-1.73E-02	>0.001

Table S1.8 Stats to Fig 7a: Ants walk straighter on fewer footprints, even within most pixels of initial straightness

st_bin	Estimate	SE	tStat	n	pValue
>= 0	-0.00138	2.76E-05	-49.7789	88931	<0.001
>=0.47	-3.87E-05	1.33E-05	-2.90034	88931	0.004
>=0.58	-2.77E-05	8.42E-06	-3.29001	88931	0.001
>=0.64	-1.67E-05	6.44E-06	-2.59618	88931	0.009
>=0.68	-2.71E-07	5.16E-06	-0.0525	88931	0.958
>=0.72	-7.10E-06	4.33E-06	-1.64121	88932	0.101
>=0.75	-9.98E-06	3.85E-06	-2.5911	88931	0.010
>=0.78	-4.10E-06	3.63E-06	-1.13183	88931	0.258
>= 0.8	-4.33E-06	3.37E-06	-1.28658	88931	0.198
>=0.82	-3.53E-06	3.26E-06	-1.08203	88931	0.279
>=0.84	-4.84E-06	3.08E-06	-1.57433	88932	0.115
>=0.86	-8.11E-06	2.96E-06	-2.74168	88931	0.006
>=0.88	-9.39E-06	2.95E-06	-3.18402	88931	0.001
>= 0.9	-1.20E-05	2.98E-06	-4.02014	88931	<0.001
>=0.91	-6.35E-06	3.01E-06	-2.11113	88931	0.035
>=0.92	-8.13E-06	3.06E-06	-2.6568	88932	0.008
>=0.94	-6.69E-06	3.05E-06	-2.19004	88931	0.029
>=0.95	-5.41E-06	3.32E-06	-1.62821	88931	0.103
>=0.96	-1.68E-05	3.64E-06	-4.61887	88931	<0.001
>=0.98	-3.11E-05	5.59E-06	-5.56502	88931	<0.001

Table S1.9 Stats to Fig 7b: Ants walk faster on fewer footprints, even within some pixels of initial straightness

v_bin	Estimate	SE	tStat	n	pValue
>= 0	-0.00547	0.000231	-23.6341	90190	<0.001
>= 2.32	-0.00125	0.000137	-9.13966	90190	<0.001
>= 5.32	-0.0007	0.000101	-6.93707	90189	<0.001
>= 6.14	-0.00037	8.13E-05	-4.51826	90190	<0.001
>= 6.74	-0.00012	7.05E-05	-1.74472	90189	0.081
>= 7.22	-0.00019	6.32E-05	-2.93028	90190	0.003
>= 7.64	-0.00016	5.92E-05	-2.76869	90189	0.006
>= 8.02	-5.53E-05	5.77E-05	-0.95875	90190	0.338
>= 8.38	6.91E-05	5.74E-05	1.204372	90189	0.228
>= 8.72	-2.36E-05	5.80E-05	-0.40767	90190	0.684
>= 9.07	4.33E-06	5.99E-05	0.072312	90190	0.942
>= 9.42	3.13E-06	6.29E-05	0.049809	90189	0.960
>= 9.78	0.00011	6.89E-05	1.594106	90190	0.111
>=10.18	3.84E-05	7.59E-05	0.505809	90189	0.613
>=10.62	6.03E-06	8.55E-05	0.070431	90190	0.944
>= 11.1	-5.02E-05	9.67E-05	-0.51929	90189	0.604
>=11.65	-2.95E-05	0.000117	-0.25133	90190	0.802
>= 12.3	-5.44E-05	0.000162	-0.33479	90189	0.738
>=13.17	-0.00039	0.000247	-1.57579	90190	0.115

>=14.45	0.026497	0.001	26.49587	90189	<0.001
