# Supplementary material for: Collective search in ants: Movement determines footprints, and footprints influence movement
Source: PLoS One. 2024 Apr 23;19(4):e0299432. doi: 10.1371/journal.pone.0299432 (PMC11037541; doi:10.1371/journal.pone.0299432)
Supplement: S2 File — All main-text analyses for an assumed 5 minute ‘evaporation’ time. (PDF) [file pone.0299432.s002.pdf]

S2: 5 min ‘evaporation’ time

Table S2.1. Ants move straighter and slower further from the nest (fig S2.3 a-d).

Name	Estimate	SE	tStat	DF	p	Lower	Upper
{'ST~FP' }	-0.0107	5.81e-05	-184	1.14e+06	<0.01	-0.0108	-0.0106
{'v~FP' }	-0.0946	0.000984	-96.1	1.15e+06	<0.01	-0.0965	-0.0927
{'ST~nestDisp'}	4.12e-05	2.90e-07	142	1.14e+06	<0.01	4.06e-05	4.18e-05
{'v~nestDisp' }	0.000359	5.02e-06	71.7	1.15e+06	<0.01	0.00035	0.000369

Table S2.2. Number of tracks whose movement correlates significantly with footprint concentration (numbers to fig S2.3 e-f).

	Straightness	Speed
Less straight/slower on higher footprint concentrations	32.67%	21.75%
Straighter/faster on higher footprint concentrations	14.8%	2.44%

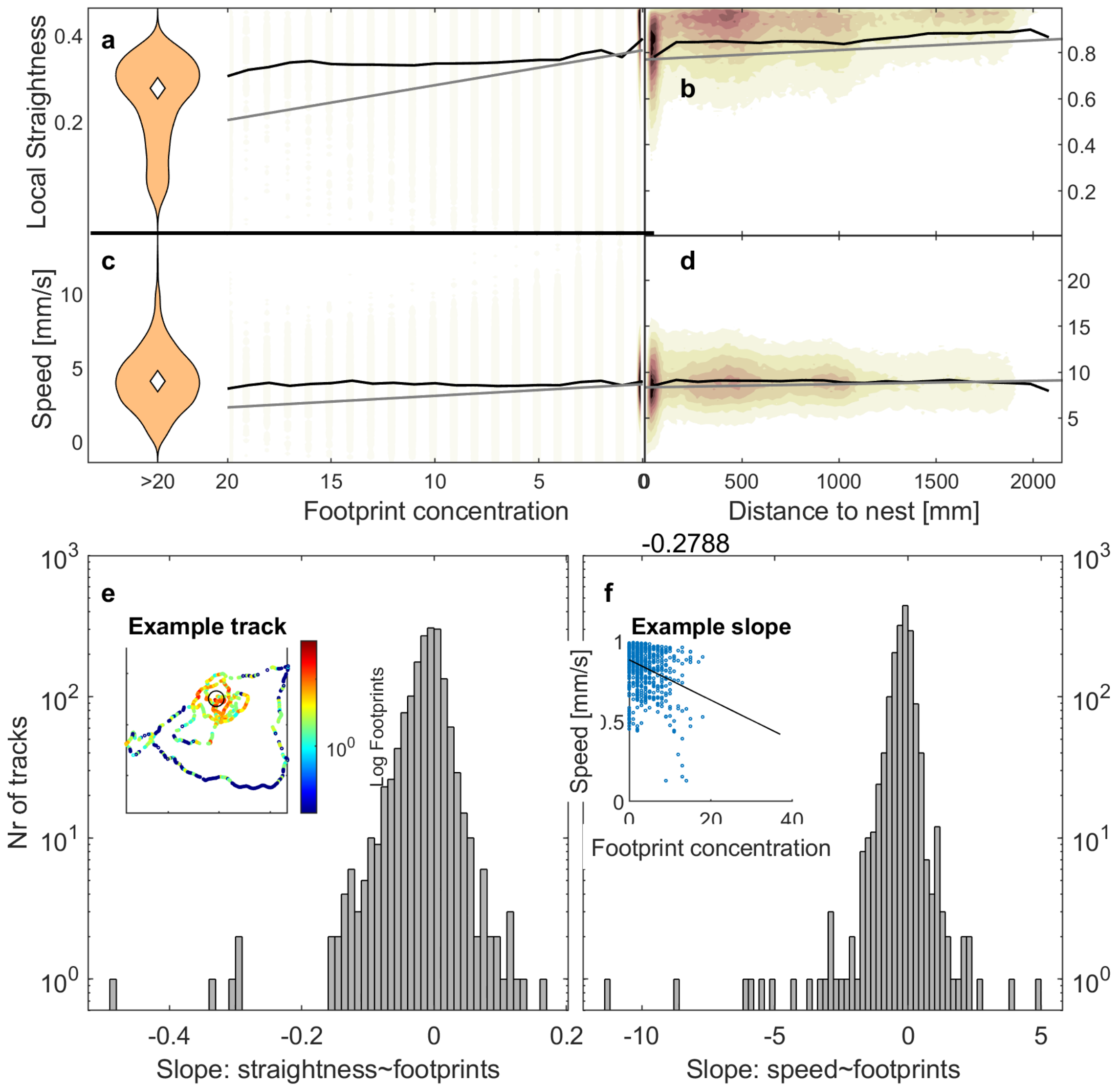

fig S2.3 *5 min 'evaporation' version of main text figures 1 and 2. a), c) Straightness and speed increase with decreasing footprint concentration. b), d) Straightness and speed increases with distance to the nest. Black lines are medians per footprint and nest distance bins, respectively. Gray lines are regressions from the LMMs. Statistical tests were performed on unbinned data. Note that the x-axes of the footprint concentration panels are reversed to be consistent with the 'Distance to the nest' plots, as the highest footprint concentrations are around the nest (i.e. towards the left side on both graphs). The majority of ants walk e) straighter and f) faster on lower footprint concentrations (see tab S2.2 for numbers). Histograms are of the slopes (for each track) of the linear models of straightness or speed ~ footprint concentration. Negative values mean ants are walking less straight or slower with increasing footprint concentration. Left inset: example track, colored by the footprint concentration the ant is currently walking over. Black open circle indicates the nest location. Right inset: scatterplot with slope of the linear model of straightness~footprints for that track.*

Table S2.4. Ants which start out straighter and slower near the nest move farther (fig S2.5 e-f).

meanDisp~stNearTheNest

	Estimate	SE	tStat	pValue
st_intrcpt	-187.17	61.284	-3.0541	0.002
st_slope	624.05	82.051	7.6056	<0.001
v_intrcpt	424.09	30.999	13.681	<0.001
v_slope	-18.52	3.7245	-4.9726	<0.001

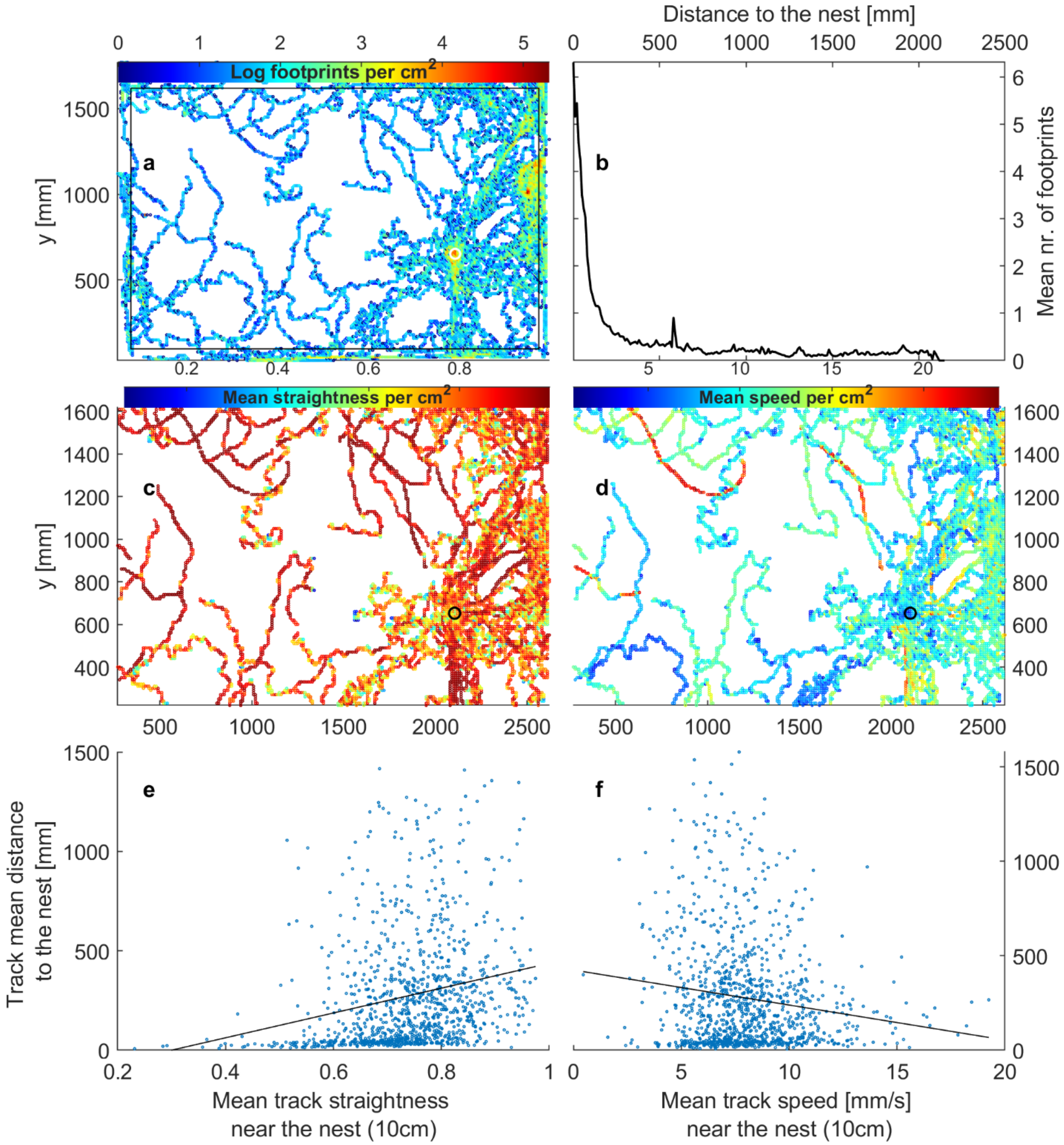

***Fig S2.5**. 5 min 'evaporation' version of main text figures 3 and 4. a), c), d): Heatmaps of the last 5 min of the experiment. a) ant visitation, binned into pixels of size 1 cm. Points outside the black rectangle were omitted from analysis. Only data of the last hour of the experiment are included. Note that ants at different timepoints experience a slightly different footprint landscape. b) Mean footprint concentration by distance to the nest. The y-axis here is the x-axis in fig 1a, x-axis here is x-axis in fig 1b). This illustrates the mismatch between the x-axes in fig 1. c) & d) heatmaps of mean straightness and speed per pixel, respectively. In a), c) & d) white and black circles indicate nest locations. e) Tracks which start out straighter move on average farther away from the nest (where there are fewer footprints), but f) tracks which are faster near the nest move less far from the nest than those which are slower near the nest.*

Table S2.6. Lower straightness on higher footprint concentration irrespective of distance to the nest. pCorr stands for Benjamini-Hochberg corrected p-values. (fig S3.12 a)

Nest dist.	slope	SE	tStat	DF	pValue	Lower	Upper	pCorr
Bin 1	-1.38E-03	1.84E-04	-7.46	47212	8.65E-14	-1.74E-03	-1.01E-03	<0.001
Bin 2	-9.95E-04	2.51E-04	-3.96	56708	7.46E-05	-1.49E-03	-5.03E-04	<0.001
Bin 3	-1.28E-02	4.66E-04	-27.4	57317	2.81E-164	-1.37E-02	-1.19E-02	<0.001
Bin 4	-2.06E-02	5.86E-04	-35.11	57276	3.73E-267	-2.17E-02	-1.94E-02	<0.001
Bin 5	-2.61E-02	6.57E-04	-39.64	57211	0.00E+00	-2.73E-02	-2.48E-02	<0.001
Bin 6	-2.52E-02	7.02E-04	-35.88	57226	9.14E-279	-2.66E-02	-2.38E-02	<0.001
Bin 7	-2.58E-02	7.44E-04	-34.6	57338	1.22E-259	-2.72E-02	-2.43E-02	<0.001
Bin 8	-3.17E-02	6.89E-04	-45.93	57389	0.00E+00	-3.30E-02	-3.03E-02	<0.001
Bin 9	-2.64E-02	6.52E-04	-40.48	57299	0.00E+00	-2.76E-02	-2.51E-02	<0.001
Bin 10	-1.99E-02	3.29E-04	-60.56	57374	0.00E+00	-2.06E-02	-1.93E-02	<0.001
Bin 11	-4.41E-02	8.35E-04	-52.86	57254	0.00E+00	-4.58E-02	-4.25E-02	<0.001
Bin 12	-6.64E-03	7.74E-05	-85.8	57449	0.00E+00	-6.79E-03	-6.49E-03	<0.001
Bin 13	-4.11E-02	7.95E-04	-51.7	57483	0.00E+00	-4.27E-02	-3.96E-02	<0.001
Bin 14	-3.26E-02	8.95E-04	-36.45	57366	1.52E-287	-3.44E-02	-3.09E-02	<0.001
Bin 15	-3.91E-02	8.89E-04	-44	57641	0.00E+00	-4.09E-02	-3.74E-02	<0.001
Bin 16	-4.29E-02	1.05E-03	-41.01	57544	0.00E+00	-4.49E-02	-4.08E-02	<0.001
Bin 17	-4.01E-02	8.97E-04	-44.66	57576	0.00E+00	-4.18E-02	-3.83E-02	<0.001
Bin 18	-5.65E-02	1.01E-03	-55.83	57673	0.00E+00	-5.85E-02	-5.45E-02	<0.001
Bin 19	-5.33E-02	9.99E-04	-53.36	57630	0.00E+00	-5.52E-02	-5.13E-02	<0.001
Bin 20	-2.81E-02	9.49E-04	-29.61	57667	3.03E-191	-3.00E-02	-2.62E-02	<0.001

Table S2.7 Mostly lower speed on higher footprint concentration across distances to the nest. pCorr stands for Benjamini-Hochberg corrected p-values. (fig S3.12 a)

Nest dist.	slope	SE	tStat	DF	pValue	Lower	Upper	pCorr
Bin 1	2.30E-02	3.11E-03	7.40	57212	1.36E-13	1.69E-02	2.91E-02	<0.001
Bin 2	-3.51E-02	4.67E-03	-7.51	57671	5.83E-14	-4.42E-02	-2.59E-02	<0.001
Bin 3	-1.85E-01	8.38E-03	-22.12	57700	5.90E-108	-2.02E-01	-1.69E-01	<0.001
Bin 4	-9.23E-02	1.04E-02	-8.91	57707	5.37E-19	-1.13E-01	-7.20E-02	<0.001

Bin 5	-1.75E-01	1.13E-02	-15.43	57714	1.23E-53	-1.97E-01	-1.53E-01	<0.001
Bin 6	-2.03E-01	1.27E-02	-16.00	57708	1.66E-57	-2.28E-01	-1.78E-01	<0.001
Bin 7	-1.78E-01	1.37E-02	-13.01	57710	1.26E-38	-2.05E-01	-1.51E-01	<0.001
Bin 8	-1.19E-01	1.22E-02	-9.73	57717	2.36E-22	-1.43E-01	-9.51E-02	<0.001
Bin 9	-1.05E-01	1.17E-02	-8.98	57718	2.80E-19	-1.27E-01	-8.18E-02	<0.001
Bin 10	6.54E-02	5.58E-03	11.71	57716	1.29E-31	5.44E-02	7.63E-02	<0.001
Bin 11	-1.86E-01	1.38E-02	-13.47	57715	2.88E-41	-2.13E-01	-1.59E-01	<0.001
Bin 12	-6.41E-02	1.31E-03	-48.71	57715	0.00E+00	-6.66E-02	-6.15E-02	<0.001
Bin 13	-9.47E-02	1.31E-02	-7.25	57723	4.35E-13	-1.20E-01	-6.91E-02	<0.001
Bin 14	-2.20E-01	1.45E-02	-15.19	57720	4.91E-52	-2.48E-01	-1.91E-01	<0.001
Bin 15	-3.68E-01	1.44E-02	-25.65	57730	3.07E-144	-3.97E-01	-3.40E-01	<0.001
Bin 16	-2.18E-01	1.82E-02	-11.94	57728	7.69E-33	-2.54E-01	-1.82E-01	<0.001
Bin 17	-1.23E-01	1.62E-02	-7.58	57724	3.50E-14	-1.55E-01	-9.11E-02	<0.001
Bin 18	-2.02E-01	1.83E-02	-10.99	57732	4.61E-28	-2.38E-01	-1.66E-01	<0.001
Bin 19	-1.60E-01	1.86E-02	-8.60	57729	8.38E-18	-1.96E-01	-1.23E-01	<0.001
Bin 20	-1.39E-01	1.76E-02	-7.90	57732	2.85E-15	-1.74E-01	-1.05E-01	<0.001

Table S2.8. Spots where ants move less straight to begin with will accumulate more footprints (stats to fig S2.12c). Supplementary to this: LMM formula: cMax ~ straightness_of_1st_point + (1|col) + (1|nestDisp): Estimate = -2.51, SE = 0.014, t = -179.7, DF = 1.138e6, p <0.001, Lower = -2.54, Upper = -2.48

Nest dist.	slope	SE	tStat	DF	pValue	Lower	Upper	pCorr
bin 01	-2.31	0.16101	-14.35	45184	<0.001	-2.63	-1.99	<0.001
bin 02	-0.51	0.101887	-5.00	55924	<0.001	-0.71	-0.31	<0.001
bin 03	-1.63	0.048688	-33.53	57390	<0.001	-1.73	-1.54	<0.001
bin 04	-1.23	0.037017	-33.13	57293	<0.001	-1.30	-1.15	<0.001
bin 05	-1.27	0.033214	-38.11	57231	<0.001	-1.33	-1.20	<0.001
bin 06	-1.15	0.032091	-35.98	57238	<0.001	-1.22	-1.09	<0.001
bin 07	-0.91	0.029259	-31.12	57375	<0.001	-0.97	-0.85	<0.001
bin 08	-1.29	0.033452	-38.43	57407	<0.001	-1.35	-1.22	<0.001
bin 09	-1.46	0.037591	-38.71	57355	<0.001	-1.53	-1.38	<0.001
bin 10	-4.93	0.099721	-49.42	57362	<0.001	-5.12	-4.73	<0.001
bin 11	-1.32	0.027647	-47.81	57291	<0.001	-1.38	-1.27	<0.001
bin 12	-0.77	0.023703	-32.68	57157	<0.001	-0.82	-0.73	<0.001
bin 13	-1.52	0.027725	-54.79	57486	<0.001	-1.57	-1.46	<0.001
bin 14	-1.17	0.024355	-48.09	57370	<0.001	-1.22	-1.12	<0.001
bin 15	-1.45	0.024941	-58.17	57652	<0.001	-1.50	-1.40	<0.001
bin 16	-0.94	0.020744	-45.19	57542	<0.001	-0.98	-0.90	<0.001
bin 17	-1.26	0.025339	-49.90	57576	<0.001	-1.31	-1.21	<0.001
bin 18	-1.19	0.021195	-56.30	57673	<0.001	-1.23	-1.15	<0.001
bin 19	-1.18	0.02097	-56.33	57619	<0.001	-1.22	-1.14	<0.001
bin 20	-0.80	0.022375	-35.83	57670	<0.001	-0.85	-0.76	<0.001

Table S2.9. Spots where ants move slower to begin with will accumulate more footprints (stats to fig S2.12d). Supplementary: LMM formula: cMax ~ speed_of_1st_point + (1|col) + (1|nestDisp): Estimate = -0.07, SE = 8.3e-4, t = -92, DF = 1.15e6, p <0.001, Lower = -0.08, Upper = -0.08.

Nest dist.	slope	SE	tStat	DF	pValue	Lower	Upper	pCorr
bin 01	-1.10E-01	7.16E-03	-15.30	56883	<0.001	-1.24E-01	-9.56E-02	<0.001
bin 02	-4.00E-03	5.21E-03	-0.77	57637	0.443	-1.42E-02	6.22E-03	0.443
bin 03	-4.92E-02	2.78E-03	-17.71	57705	<0.001	-5.47E-02	-4.38E-02	<0.001
bin 04	-2.80E-02	2.09E-03	-13.44	57711	<0.001	-3.21E-02	-2.39E-02	<0.001
bin 05	-3.91E-02	1.92E-03	-20.37	57716	<0.001	-4.29E-02	-3.53E-02	<0.001
bin 06	-3.83E-02	1.78E-03	-21.55	57714	<0.001	-4.18E-02	-3.49E-02	<0.001
bin 07	-2.78E-02	1.59E-03	-17.43	57711	<0.001	-3.09E-02	-2.46E-02	<0.001
bin 08	-3.24E-02	1.90E-03	-17.08	57718	<0.001	-3.61E-02	-2.87E-02	<0.001
bin 09	-5.28E-02	2.20E-03	-23.94	57718	<0.001	-5.71E-02	-4.85E-02	<0.001
bin 10	-6.07E-02	5.99E-03	-10.14	57714	<0.001	-7.24E-02	-4.90E-02	<0.001
bin 11	-3.57E-02	1.70E-03	-21.06	57708	<0.001	-3.91E-02	-3.24E-02	<0.001
bin 12	-3.52E-01	1.21E-02	-28.95	57567	<0.001	-3.75E-01	-3.28E-01	<0.001
bin 13	-3.56E-02	1.75E-03	-20.35	57718	<0.001	-3.91E-02	-3.22E-02	<0.001
bin 14	-3.53E-02	1.53E-03	-22.99	57720	<0.001	-3.83E-02	-3.23E-02	<0.001
bin 15	-5.22E-02	1.59E-03	-32.75	57730	<0.001	-5.53E-02	-4.91E-02	<0.001
bin 16	-2.37E-02	1.21E-03	-19.50	57729	<0.001	-2.61E-02	-2.13E-02	<0.001
bin 17	-1.65E-02	1.45E-03	-11.40	57724	<0.001	-1.93E-02	-1.37E-02	<0.001
bin 18	-2.16E-02	1.21E-03	-17.80	57732	<0.001	-2.39E-02	-1.92E-02	<0.001
bin 19	-2.13E-02	1.17E-03	-18.11	57727	<0.001	-2.36E-02	-1.90E-02	<0.001
bin 20	-1.59E-02	1.22E-03	-13.02	57731	<0.001	-1.83E-02	-1.35E-02	<0.001

Table S2.10. Ants walk straighter on fewer footprints, even within pixels of initial straightness (stats to fig S2.12e)

st_bin	Estimate	SE	tStat	n	pValue
>= 0	-0.00288	5.01E-05	-57.434	56883	<0.001
>=0.47	-0.00034	5.56E-05	-6.154	56884	<0.001
>=0.57	-4.92E-05	3.52E-05	-1.401	56884	0.161
>=0.64	-5.28E-05	2.57E-05	-2.056	56883	0.040
>=0.69	-6.79E-06	2.13E-05	-0.319	56884	0.750
>=0.72	-4.78E-05	1.88E-05	-2.545	56884	0.011
>=0.76	-2.76E-05	1.65E-05	-1.669	56883	0.095
>=0.78	-1.65E-05	1.49E-05	-1.109	56884	0.267
>=0.81	-1.70E-05	1.41E-05	-1.210	56884	0.226
>=0.83	-3.08E-05	1.32E-05	-2.329	56883	0.020
>=0.85	-9.96E-06	1.25E-05	-0.797	56884	0.426
>=0.87	-1.87E-05	1.22E-05	-1.538	56884	0.124
>=0.88	-1.05E-05	1.18E-05	-0.891	56884	0.373
>= 0.9	-1.40E-05	1.16E-05	-1.208	56883	0.227

>=0.91	-1.34E-05	1.19E-05	-1.132	56884	0.257
>=0.93	-1.84E-05	1.16E-05	-1.585	56884	0.113
>=0.94	-2.04E-05	1.22E-05	-1.677	56883	0.093
>=0.95	-2.44E-05	1.32E-05	-1.848	56884	0.065
>=0.97	-4.73E-05	1.41E-05	-3.366	56884	<0.001
>=0.98	-6.77E-05	2.10E-05	-3.218	56883	0.001

Table S2.11. Ants walk straighter on fewer footprints, even within pixels of initial straightness (stats to fig S2.12f)

v_bin	Estimate	SE	tStat	n	pValue
>= 0	-0.01184	0.000481	-24.611	57694	<0.001
>= 2.32	-0.0034	0.000463	-7.337	57693	<0.001
>= 5.27	-0.00196	0.000408	-4.809	57693	<0.001
>= 6.08	-0.00022	0.000338	-0.654	57693	0.513
>= 6.66	-0.00016	0.000309	-0.529	57693	0.597
>= 7.14	-0.00056	0.000256	-2.178	57693	0.029
>= 7.55	-0.00022	0.000238	-0.905	57693	0.365
>= 7.93	0.000131	0.000248	0.526	57693	0.599
>= 8.28	8.77E-05	0.000239	0.368	57693	0.713
>= 8.63	-0.0002	0.000239	-0.824	57693	0.410
>= 8.96	-0.00014	0.000239	-0.587	57693	0.557
>= 9.31	0.000136	0.000251	0.544	57693	0.586
>= 9.67	0.000415	0.000267	1.554	57693	0.120
>=10.06	-0.0003	0.000293	-1.026	57693	0.305
>=10.49	-0.00011	0.000306	-0.356	57693	0.722
>=10.96	0.000774	0.000359	2.158	57693	0.031
>= 11.5	0.000573	0.000409	1.402	57693	0.161
>=12.13	0.001214	0.000526	2.308	57693	0.021
>=12.96	-0.00032	0.00073	-0.436	57693	0.663
>=14.19	0.045151	0.002578	17.517	57693	<0.001

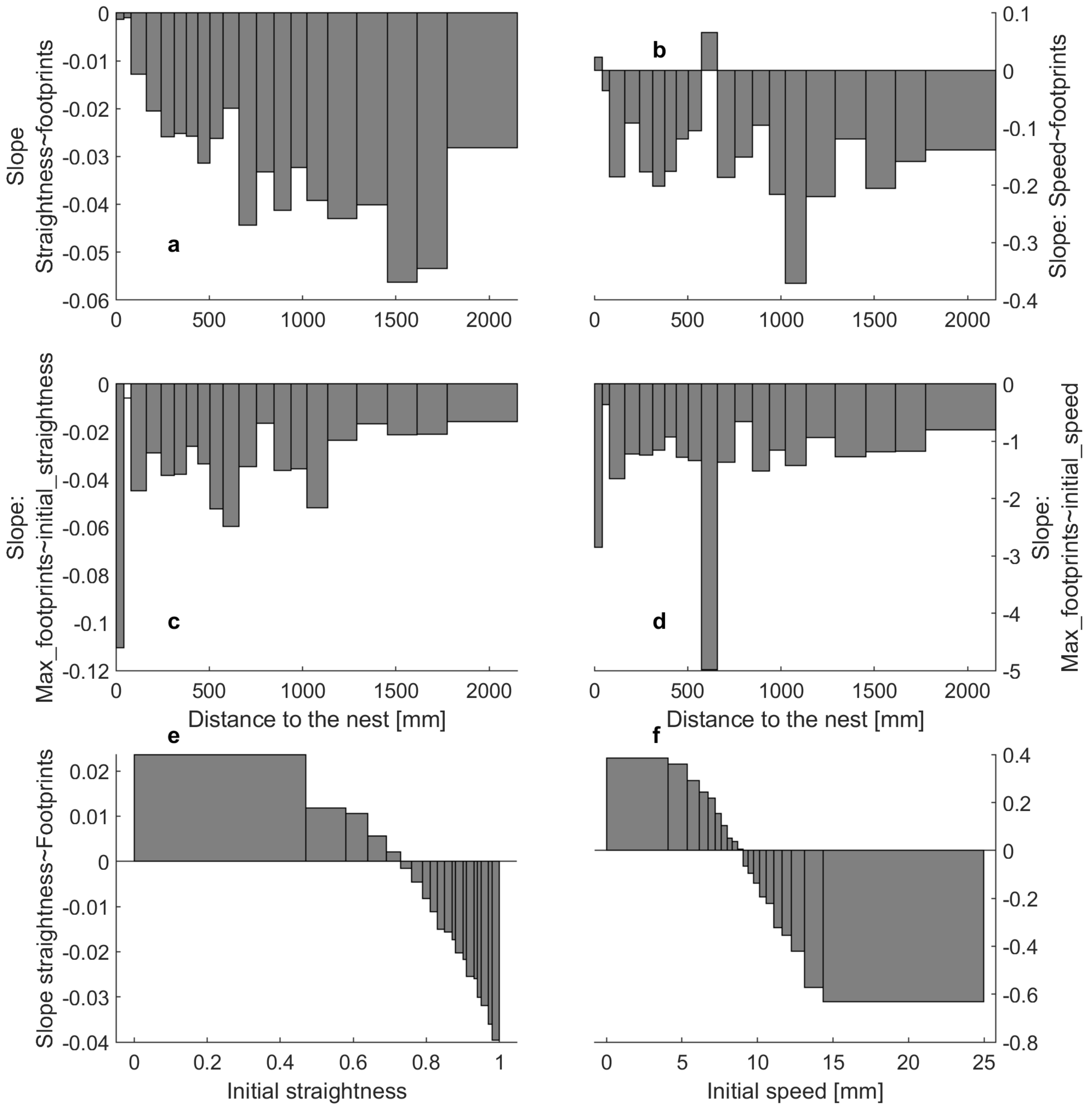

Fig S2.12. *5 min 'evaporation' version of main text figures 5, 6, and 7. Ants walk a) straighter and b) mostly faster with lower footprint concentration, even when binning by (= controlling for) distance to the nest. Bins are sized to each contain about 9.04x10^4 points. c) Slopes of mean final footprint number of 2x2 mm pixels over the straightness value of the first point created in that pixel, for each nest distance bin. d) Same as c), but with speed on the x-axis. e) Slopes of the linear model of (straightness~footprints) over the straightness of the first point in the respective pixel. Negative values indicate ants walking straighter on lower footprint concentrations. f) same as e) for initial speed. Filled bars (i.e., almost all bars) indicate values significantly different from 0.*

Ants do not turn preferentially in the direction of higher or lower footprint concentrations (Fig S2.13; LMM: turn_angle ~ L-R, Estimate = -0.026, SE = 0.026, t = -0.977, n = 293292, p = 0.329].

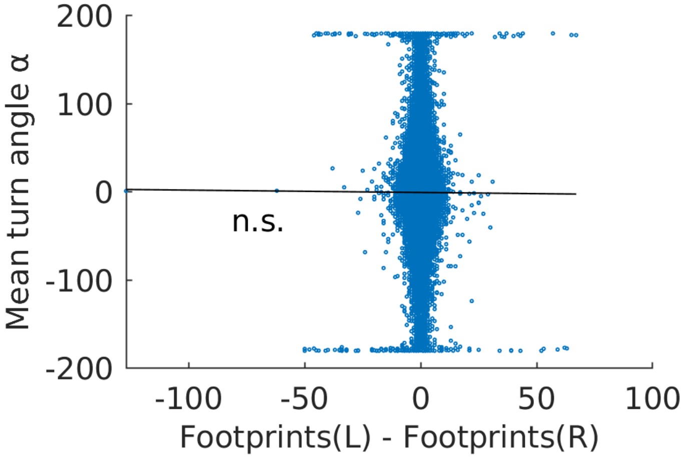

Fig. S2.13. *5 min evaporation version of main text figure 8. Ants do not turn toward or away from a footprint gradient. A negative slope would indicate turning toward the side of higher footprint concentrations.*
