# Supplementary material for: Collective search in ants: Movement determines footprints, and footprints influence movement
Source: PLoS One. 2024 Apr 23;19(4):e0299432. doi: 10.1371/journal.pone.0299432 (PMC11037541; doi:10.1371/journal.pone.0299432)
Supplement: S3 File — All main-text analyses for an assumed 5 h ‘evaporation’ time. (PDF) [file pone.0299432.s003.pdf]

S3: No ‘evaporation’ time

All points are counted toward the footprint concentration, regardless of time since creation. This is equivalent to an evaporation time of at least 5h.

Table S3.1. Ants move straighter and slower further from the nest (fig S3.3 a-d). LMMs with colony as ‘random factor’.

**Name**	**Estimate**	**SE**	**tStat**	**DF**	**pValue**	**Lower**	**Upper**
st~c	-3.8e-03	2.539e-05	-149.8	1.916e+06	<0.001	-3.853e-03	-3.754e-03
v~c	-0.0217	4.564e-04	-47.489	1.916e+06	<0.001	-0.0226	-0.02078
st~nestDisp	2.588e-05	2.325e-07	111.3	1.916e+06	<0.001	2.542e-05	2.633e-05
v~nestDisp	1.94e-04	4.229e-06	45.858	1.916e+06	<0.001	1.857e-04	2.023e-04

Table S3.2. Number of tracks whose movement correlates significantly with footprint concentration (numbers to fig S3.3 e-f).

	**Straightness**	**Speed**
Less straight/slower on higher footprint concentrations	32.3%	18.0%
Straighter/faster on higher footprint concentrations	17.3%	3.1%

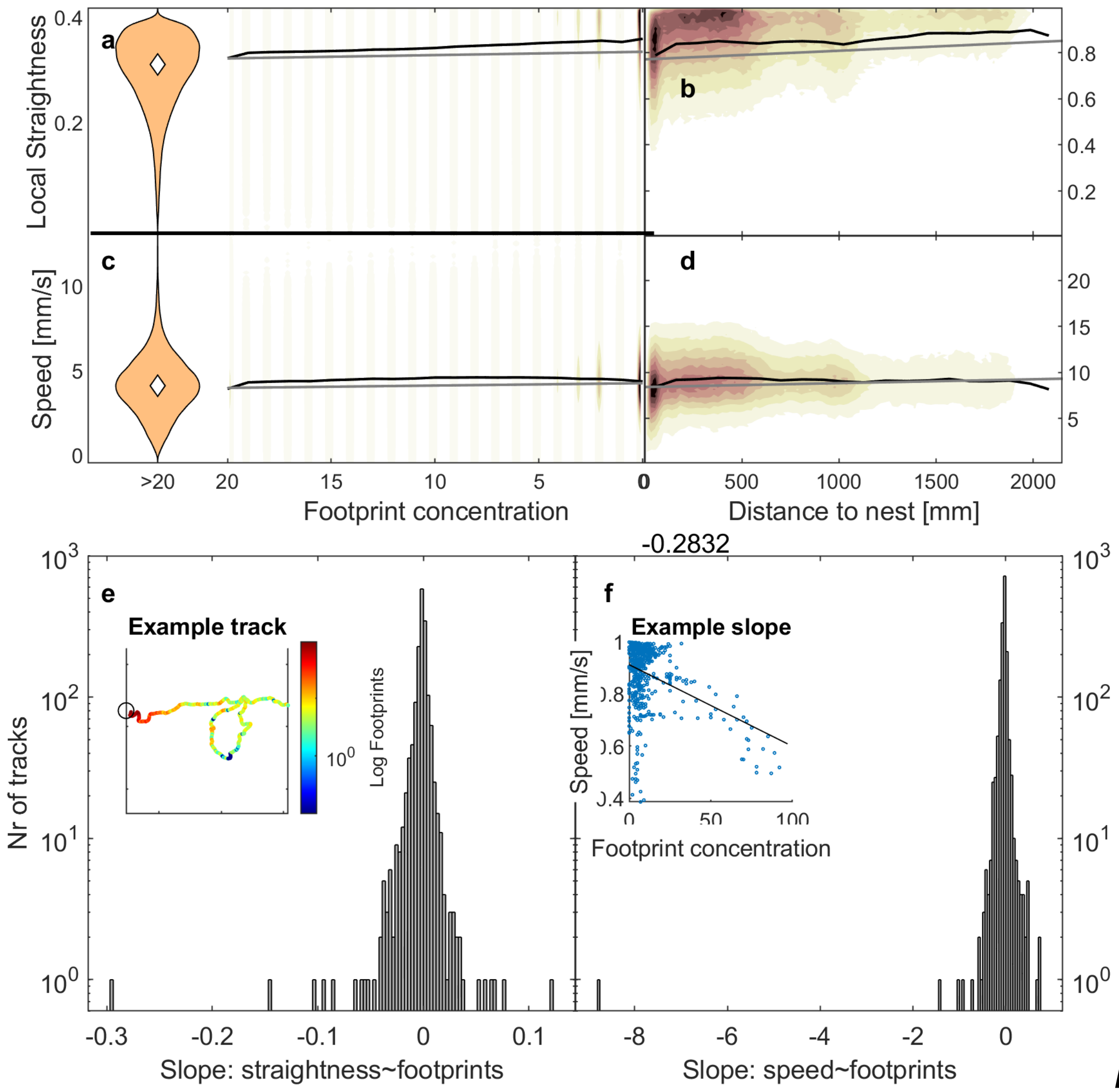

Fig S3.3*.* *5h 'evaporation' version of the main text figures 1 and 2. a), c) Straightness and speed increase with decreasing footprint concentration. b), d) Straightness and speed increases with distance to the nest. Black lines are medians per footprint and nest distance bins, respectively. Gray lines are regressions from the LMMs. Statistical tests were performed on unbinned data. Note that the x-axis of the footprint concentration panels are reversed to be consistent with the 'Distance to the nest' plots, as the highest footprint concentrations are around the nest (i.e. towards the left side on both graphs). More ants walk significantly e) straighter and f) faster on lower footprint concentrations than the opposite (see tab S3.2 for numbers). Histograms are of the slopes (for each track) of the linear models of straightness or speed ~ footprint concentration. Negative values mean ants are walking less straight or slower with increasing footprint concentration. Left inset: example track, colored by the footprint concentration the ant is currently walking over. Black open circle indicates the nest location. Right inset: scatterplot with slope of the linear model of straightness~footprints for that track.*

We indeed found that ants that start out straighter near the nest move farther away from it [LM mean(dist)~ST_near_nest: SE = 77.93, t = 9.83, p < .001; fig S3.5e]. We found the opposite

effect on speed [LM mean(dist)~v_near_nest: SE = 28.74, t = 12.36, $p < 0.001$; Fig 4b]. Both results confirm that the high straightness and low speed of ants far away from the nest may be due to those particular ants having those characteristics already on leaving the nest, rather than changing their behavior as they gain distance from the nest.

Table S3.4. Ants which start out straighter and slower near the nest move farther (fig S3.5 e-f).

	**Estimate**	**SE**	**tStat**	**pValue**
st_intrcpt	-311.115	58.943	-5.278	<0.001
st_slope	765.833	77.931	9.827	<0.001
v_intrcpt	355.174	28.735	12.360	<0.001
v_slope	-10.948	3.342	-3.276	0.001

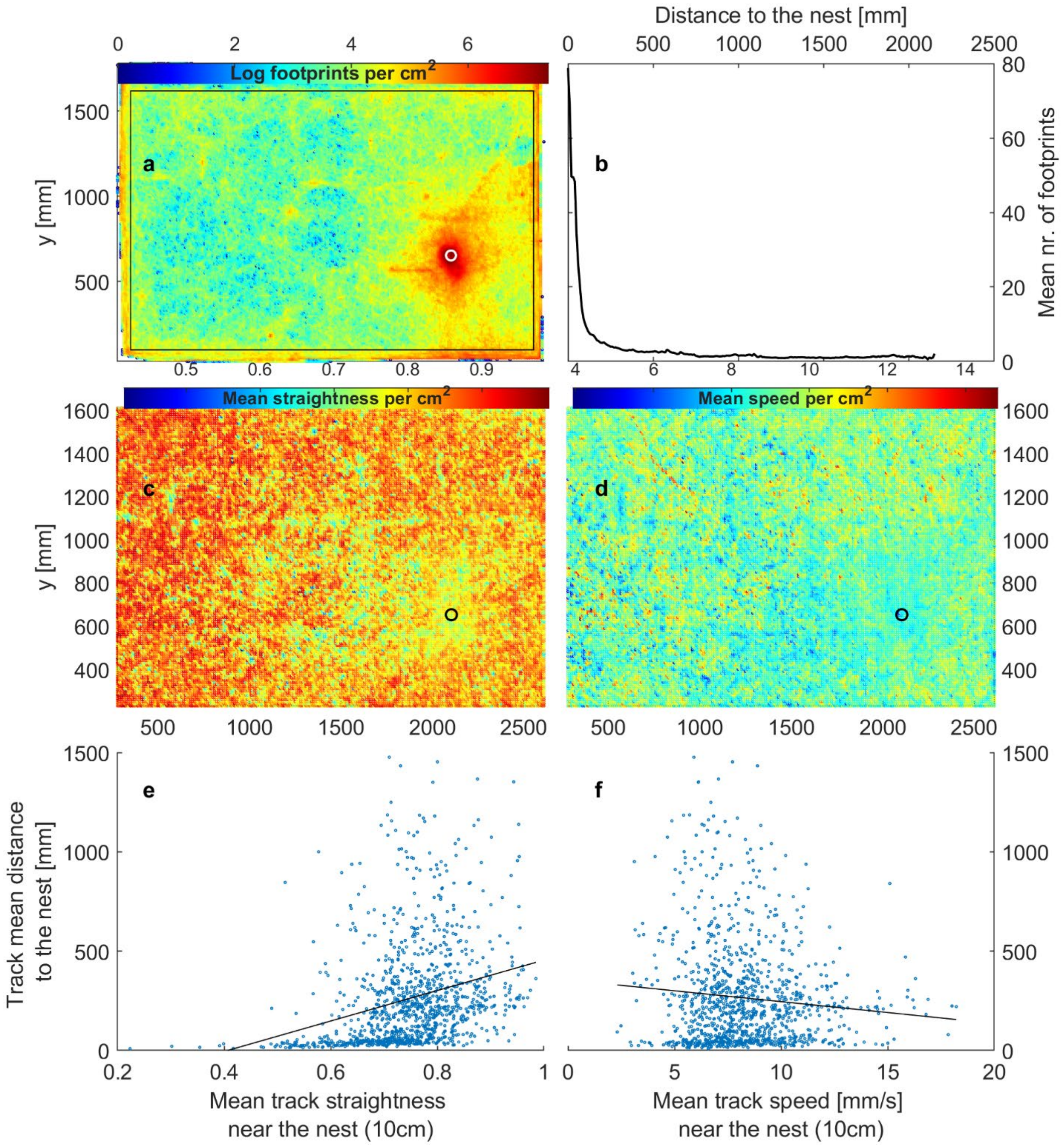

Fig S3.5. *5 h 'evaporation' version of main text figures 3 and 4. a), c), d): Heatmaps of the last 5 min of the experiment. a) ant visitation, binned into pixels of size 1 cm. Points outside the black rectangle were omitted from analysis. Only data of the last hour of the experiment are included. Note that ants at different timepoints experience a slightly different footprint landscape. b) Mean footprint concentration by distance to the nest. The y-axis here is the x-axis in fig 1a, x-axis here is x-axis in fig 1b). This illustrates the mismatch between the x-axes in fig 1. c) & d) heatmaps of mean straightness and speed per pixel, respectively. In a), c) & d) white and black circles indicate nest locations. e) Tracks which start out straighter move on average farther away from the nest (where there are fewer footprints), but f) tracks which are faster near the nest move less far from the nest than those which are slower near the nest.*

Table S3.6 Lower straightness on higher footprint concentration irrespective of distance to the nest. pCorr stands for Benjamini-Hochberg corrected p-values. (fig S3.12 a)

Name	slope	SE	tStat	DF	pValue	Lower	Upper	pCorr
ST bin1	-3.100E-04	1.740E-05	-17.985	92810	<0.001	-3.500E-04	-2.800E-04	<0.001
ST bin2	-3.800E-04	2.700E-05	-13.929	107428	<0.001	-4.300E-04	-3.200E-04	<0.001
ST bin3	-9.900E-04	6.470E-05	-15.328	108019	<0.001	-1.120E-03	-8.600E-04	<0.001
ST bin4	-2.760E-03	8.520E-05	-32.394	108119	<0.001	-2.930E-03	-2.590E-03	<0.001
ST bin5	-2.460E-03	1.040E-04	-23.576	107515	<0.001	-2.670E-03	-2.260E-03	<0.001
ST bin6	-3.360E-03	1.220E-04	-27.428	107536	<0.001	-3.600E-03	-3.120E-03	<0.001
ST bin7	-3.330E-03	1.400E-04	-23.804	107688	<0.001	-3.600E-03	-3.050E-03	<0.001
ST bin8	-4.300E-03	1.310E-04	-32.901	107893	<0.001	-4.560E-03	-4.050E-03	<0.001
ST bin9	-3.190E-03	1.480E-04	-21.555	108013	<0.001	-3.480E-03	-2.900E-03	<0.001
ST bin10	-4.840E-03	1.440E-04	-33.596	107953	<0.001	-5.120E-03	-4.550E-03	<0.001
ST bin11	-6.540E-03	1.100E-04	-59.498	107941	<0.001	-6.750E-03	-6.320E-03	<0.001
ST bin12	-5.140E-03	1.420E-04	-36.137	107664	<0.001	-5.420E-03	-4.860E-03	<0.001
ST bin13	-5.690E-03	1.800E-04	-31.669	107703	<0.001	-6.040E-03	-5.340E-03	<0.001
ST bin14	-3.960E-03	5.080E-05	-78.055	108084	<0.001	-4.060E-03	-3.860E-03	<0.001
ST bin15	-5.930E-03	2.040E-04	-29.108	108008	<0.001	-6.330E-03	-5.530E-03	<0.001
ST bin16	-7.190E-03	1.880E-04	-38.303	108458	<0.001	-7.560E-03	-6.830E-03	<0.001
ST bin17	-9.000E-03	2.500E-04	-36.021	108379	<0.001	-9.490E-03	-8.510E-03	<0.001
ST bin18	-4.710E-03	2.520E-04	-18.643	108427	<0.001	-5.200E-03	-4.210E-03	<0.001
ST bin19	-8.680E-03	2.650E-04	-32.777	108513	<0.001	-9.200E-03	-8.160E-03	<0.001
ST bin20	-2.060E-03	2.170E-04	-9.490	108553	<0.001	-2.490E-03	-1.640E-03	<0.001

Table S3.7 Mostly lower speed on higher footprint concentration across distances to the nest. pCorr stands for Benjamini-Hochberg corrected p-values. (fig S3.12 b)

Name	slope	SE	tStat	DF	pValue	Lower	Upper	pCorr
v bin1	-1.825E-02	2.750E-04	-66.403	107891	<0.001	-1.879E-02	-1.771E-02	<0.001
v bin2	-1.942E-02	5.000E-04	-38.885	108603	<0.001	-2.040E-02	-1.844E-02	<0.001
v bin3	-2.591E-02	1.224E-03	-21.164	108630	<0.001	-2.831E-02	-2.351E-02	<0.001
v bin4	-4.094E-02	1.641E-03	-24.941	108628	<0.001	-4.415E-02	-3.772E-02	<0.001
v bin5	-8.140E-03	1.965E-03	-4.142	108624	<0.001	-1.199E-02	-4.290E-03	<0.001
v bin6	-1.581E-02	2.281E-03	-6.934	108627	<0.001	-2.028E-02	-1.134E-02	<0.001
v bin7	-6.000E-04	2.635E-03	-0.228	108636	0.819	-5.770E-03	4.563E-03	0.83
v bin8	-4.621E-02	2.499E-03	-18.495	108635	<0.001	-5.111E-02	-4.132E-02	<0.001
v bin9	4.802E-03	2.862E-03	1.678	108641	0.093	-8.100E-04	1.041E-02	0.104
v bin10	1.634E-02	2.678E-03	6.103	108635	<0.001	1.109E-02	2.159E-02	<0.001
v bin11	1.916E-02	2.010E-03	9.534	108645	<0.001	1.522E-02	2.310E-02	<0.001
v bin12	2.299E-02	2.542E-03	9.042	108619	<0.001	1.800E-02	2.797E-02	<0.001
v bin13	-3.230E-02	3.066E-03	-10.535	108625	<0.001	-3.831E-02	-2.629E-02	<0.001
v bin14	-3.648E-02	8.390E-04	-43.467	108644	<0.001	-3.812E-02	-3.483E-02	<0.001
v bin15	-1.136E-02	3.419E-03	-3.322	108644	<0.001	-1.806E-02	-4.660E-03	0.001
v bin16	-5.342E-02	3.093E-03	-17.270	108661	<0.001	-5.948E-02	-4.735E-02	<0.001
v bin17	-3.804E-02	4.353E-03	-8.739	108662	<0.001	-4.657E-02	-2.951E-02	<0.001

v bin18	-4.099E-02	4.663E-03	-8.790	108659	<0.001	-5.013E-02	-3.185E-02	<0.001
v bin19	-1.010E-03	4.706E-03	-0.214	108665	0.83	-1.023E-02	8.217E-03	0.83
v bin20	2.608E-02	4.111E-03	6.345	108665	2.23E-10	1.803E-02	3.414E-02	<0.001

Table S3.8. Spots where ants move less straight to begin with will accumulate more footprints (stats to fig S3.12c). Supplementary: LMM maximum footprint number ~ initial straightness, with 'nest distance' and 'colony' as random factors: Estimate = -2.51, SE = 0.01, t = -179.7, DF = 1.14e6, p<0.001, Lower = -2.54, Upper = -2.48.

Nest dist.	slope	SE	tStat	DF	pValue	Lower	Upper	pCorr
bin 01	-3.18	0.53	-5.99	88918	<0.001	-4.21	-2.14	<0.001
bin 02	-0.36	0.37	-0.96	106321	0.338	-1.09	0.38	0.338
bin 03	-2.37	0.16	-14.68	108115	<0.001	-2.68	-2.05	<0.001
bin 04	-5.61	0.13	-42.18	108222	<0.001	-5.87	-5.35	<0.001
bin 05	-1.90	0.11	-17.01	107793	<0.001	-2.12	-1.68	<0.001
bin 06	-1.40	0.10	-14.17	107679	<0.001	-1.60	-1.21	<0.001
bin 07	-1.65	0.08	-19.39	107698	<0.001	-1.81	-1.48	<0.001
bin 08	-3.86	0.10	-40.64	107916	<0.001	-4.05	-3.68	<0.001
bin 09	-1.01	0.08	-12.42	108148	<0.001	-1.17	-0.85	<0.001
bin 10	-1.31	0.09	-15.33	108104	<0.001	-1.48	-1.15	<0.001
bin 11	-6.52	0.13	-50.17	108036	<0.001	-6.78	-6.27	<0.001
bin 12	-2.04	0.09	-22.44	107779	<0.001	-2.22	-1.86	<0.001
bin 13	-1.99	0.07	-27.49	107834	<0.001	-2.13	-1.84	<0.001
bin 14	-1.48	0.05	-28.78	107872	<0.001	-1.59	-1.38	<0.001
bin 15	-2.47	0.06	-42.23	108034	<0.001	-2.59	-2.36	<0.001
bin 16	-4.16	0.06	-65.36	108454	<0.001	-4.28	-4.03	<0.001
bin 17	-2.25	0.05	-46.30	108362	<0.001	-2.34	-2.15	<0.001
bin 18	-1.55	0.05	-33.68	108418	<0.001	-1.64	-1.46	<0.001
bin 19	-1.90	0.04	-42.86	108493	<0.001	-1.98	-1.81	<0.001
bin 20	-1.35	0.05	-25.51	108552	<0.001	-1.45	-1.24	<0.001

Table S3.9. Spots where ants move slower to begin with will accumulate more footprints (stats to fig S3.12d). Supplementary: LMM maximum footprint number ~ initial speed, with 'nest distance' and 'colony' as random factors: Estimate = -0.08, SE = 8.3e-4, t = -92, DF = 1.15e6, p<0.001, Lower = -0.08, Upper = -0.07

Nest dist.	slope	SE	tStat	DF	pValue	Lower	Upper	pCorr
bin 01	-1.37E-01	2.80E-02	-4.90	107181	<0.001	-1.92E-01	-8.23E-02	<0.001
bin 02	3.29E-01	2.09E-02	15.71	108525	<0.001	2.88E-01	3.70E-01	<0.001
bin 03	-2.69E-02	8.81E-03	-3.06	108642	0.002	-4.42E-02	-9.65E-03	0.002
bin 04	-2.12E-01	7.01E-03	-30.20	108633	<0.001	-2.25E-01	-1.98E-01	<0.001
bin 05	-8.92E-02	5.80E-03	-15.38	108645	<0.001	-1.01E-01	-7.78E-02	<0.001
bin 06	-7.75E-02	5.14E-03	-15.09	108630	<0.001	-8.76E-02	-6.74E-02	<0.001

bin 07	-2.46E-02	4.45E-03	-5.52	108626	<0.001	-3.33E-02	-1.59E-02	<0.001
bin 08	-1.85E-01	4.91E-03	-37.78	108633	<0.001	-1.95E-01	-1.76E-01	<0.001
bin 09	-6.68E-02	4.14E-03	-16.14	108645	<0.001	-7.49E-02	-5.87E-02	<0.001
bin 10	-5.62E-02	4.50E-03	-12.49	108641	<0.001	-6.50E-02	-4.74E-02	<0.001
bin 11	-2.30E-01	7.14E-03	-32.27	108651	<0.001	-2.44E-01	-2.16E-01	<0.001
bin 12	-7.18E-02	5.13E-03	-14.01	108607	<0.001	-8.19E-02	-6.18E-02	<0.001
bin 13	-9.02E-02	4.26E-03	-21.18	108631	<0.001	-9.86E-02	-8.19E-02	<0.001
bin 14	-4.18E-01	1.39E-02	-30.07	108486	<0.001	-4.45E-01	-3.91E-01	<0.001
bin 15	-9.95E-02	3.56E-03	-27.98	108632	<0.001	-1.06E-01	-9.25E-02	<0.001
bin 16	-1.49E-01	4.03E-03	-36.90	108655	<0.001	-1.57E-01	-1.41E-01	<0.001
bin 17	-9.81E-02	2.79E-03	-35.13	108659	<0.001	-1.04E-01	-9.26E-02	<0.001
bin 18	-6.44E-02	2.50E-03	-25.75	108651	<0.001	-6.93E-02	-5.95E-02	<0.001
bin 19	-5.34E-02	2.48E-03	-21.49	108658	<0.001	-5.82E-02	-4.85E-02	<0.001
bin 20	-4.81E-02	2.82E-03	-17.06	108659	<0.001	-5.36E-02	-4.26E-02	<0.001

Table S3.10. Ants walk mostly straighter on fewer footprints, even within pixels of initial straightness (stats to fig S3.12e)

**st_bin**	**Estimate**	**SE**	**tStat**	**n**	**pValue**
>= 0	-4.9E-04	1.51E-05	-32.345	107237	<0.001
>=0.48	-1.47E-05	4.98E-06	-2.956	107237	0.003
>=0.58	-7.65E-06	3.13E-06	-2.444	107237	0.015
>=0.64	-4.34E-06	2.40E-06	-1.804	107237	0.071
>=0.69	-4.45E-07	1.94E-06	-0.229	107238	0.819
>=0.72	-5.81E-06	1.66E-06	-3.506	107237	0.000
>=0.75	-2.84E-06	1.48E-06	-1.916	107237	0.055
>=0.78	-2.98E-06	1.37E-06	-2.172	107237	0.030
>= 0.8	-3.46E-06	1.30E-06	-2.669	107238	0.008
>=0.83	-5.74E-07	1.24E-06	-0.463	107237	0.644
>=0.84	-2.28E-06	1.18E-06	-1.933	107237	0.053
>=0.86	-2.00E-06	1.16E-06	-1.723	107237	0.085
>=0.88	-2.54E-06	1.15E-06	-2.212	107238	0.027
>= 0.9	-1.40E-06	1.15E-06	-1.219	107237	0.223
>=0.91	-1.65E-07	1.12E-06	-0.148	107237	0.883
>=0.93	-3.68E-06	1.14E-06	-3.218	107237	0.001
>=0.94	-1.50E-06	1.16E-06	-1.295	107238	0.195
>=0.95	2.33E-06	1.17E-06	1.988	107237	0.047
>=0.97	-4.51E-06	1.30E-06	-3.479	107237	<0.001
>=0.98	-1.20E-05	2.03E-06	-5.939	107237	<0.001

Table S3.11. Ants walk straighter on fewer footprints, even within pixels of initial straightness (stats to fig S3.12f)

**v_bin**	**Estimate**	**SE**	**tStat**	**n**	**pValue**
>= 0	-2.300E-03	1.120E-04	-20.527	108604	<0.001

>= 2.32	-5.000E-04	5.450E-05	-9.189	108604	<0.001
>= 5.38	-2.500E-04	3.930E-05	-6.304	108604	<0.001
>= 6.19	-1.300E-04	3.090E-05	-4.047	108604	<0.001
>= 6.79	-3.720E-05	2.680E-05	-1.386	108604	0.166
>= 7.27	-7.400E-05	2.460E-05	-3.009	108604	0.003
>= 7.69	-2.330E-05	2.320E-05	-1.006	108604	0.314
>= 8.07	-7.880E-07	2.220E-05	-0.035	108604	0.972
>= 8.42	6.430E-06	2.240E-05	0.287	108604	0.774
>= 8.77	-1.400E-05	2.240E-05	-0.625	108604	0.532
>= 9.11	7.020E-07	2.240E-05	0.031	108604	0.975
>= 9.46	1.240E-05	2.370E-05	0.524	108604	0.6
>= 9.83	2.610E-05	2.600E-05	1.002	108604	0.316
>=10.23	2.510E-05	2.860E-05	0.878	108604	0.380
>=10.67	8.430E-06	3.240E-05	0.260	108604	0.795
>=11.15	-2.300E-05	3.640E-05	-0.630	108604	0.529
>= 11.7	1.210E-05	4.500E-05	0.268	108604	0.788
>=12.36	-6.890E-06	6.220E-05	-0.111	108604	0.912
>=13.22	-4.810E-05	9.490E-05	-0.507	108604	0.612
>= 14.5	1.221E-02	3.410E-04	35.845	108603	<0.001

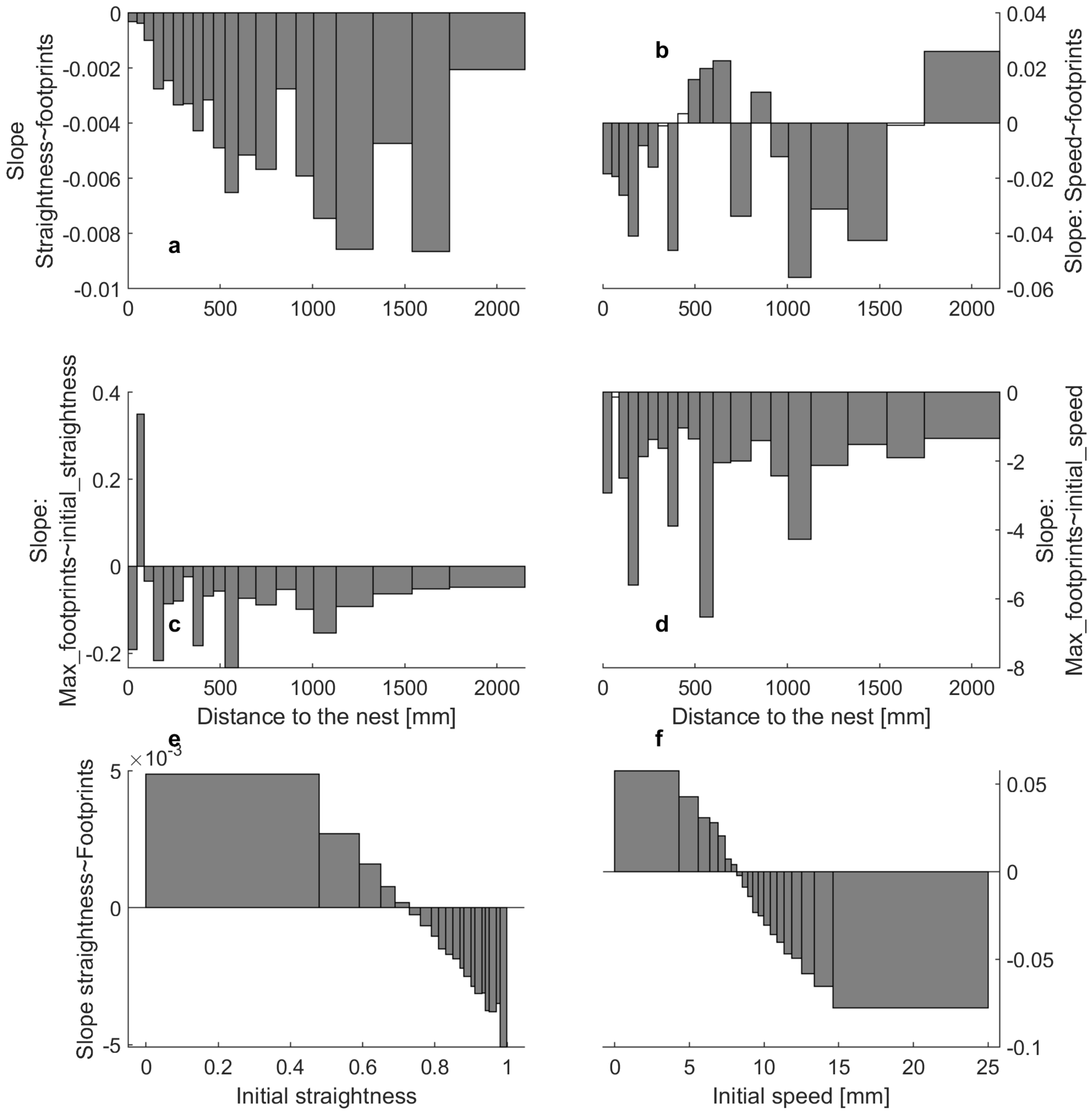

Fig S3.12. *5 h 'evaporation' version of main text figures 5, 6, and 7. Ants walk a) straighter and b) mostly faster with lower footprint concentration, even when binning by (= controlling for) distance to the nest. Bins are sized to each contain about 9.04x10^4 points. c) Slopes of mean final footprint number of 2x2 mm pixels over the straightness value of the first point created in that pixel, for each nest distance bin. d) Same as c), but with speed on the x-axis. e) slopes of the linear model of (straightness~footprints) over the straightness of the first point in the respective pixel. Negative values indicate ants walking straighter on lower footprint concentrations. f) same as e) for initial speed. Filled bars indicate values significantly different from 0.*

Ants do not turn preferentially in the direction of higher or lower footprint concentrations (Fig S3.13; LM: turn_angle ~ L-R, Estimate = -7.58e-5, SE = 5.55e-3, n = 1342685, t = -0.014, p = 0.989].

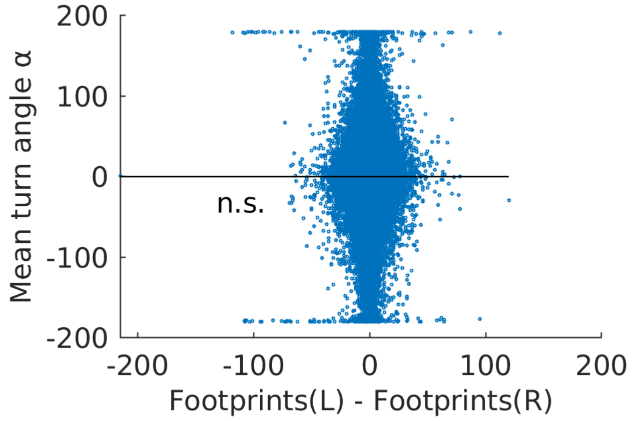

***Fig. S3.13**. 5 h evaporation version of main text figure 8. Ants do not turn toward or away from a footprint gradient. A negative slope would indicate turning toward the side of higher footprint concentrations.*
