# Supplementary material for: Collective search in ants: Movement determines footprints, and footprints influence movement
Source: PLoS One. 2024 Apr 23;19(4):e0299432. doi: 10.1371/journal.pone.0299432 (PMC11037541; doi:10.1371/journal.pone.0299432)
Supplement: S4 File — All main-text analyses on the ‘No cleaning’ data of Hunt et al. 2016. (PDF) [file pone.0299432.s004.pdf]

S4 Hunt et al. ‘NC’ (footprints present)

Table S4.1. Ants move straighter and slower further from the nest (fig S4.3 a-d). LMMs with ‘colony’ as a random factor.

Name	Estimate	SE	tStat	DF	pValue	Lower	Upper
st~c	-6.048e-03	1.396e-04	-43.329	54532	<0.001	-6.322e-03	-5.775e-03
v~c	-0.0442	2.756e-03	-16.038	54532	<0.001	-0.0496	-0.03881
st~nestDisp	3.148e-04	7.908e-06	39.803	54532	<0.001	2.993e-04	3.303e-04
v~nestDisp	5.327e-03	1.541e-04	34.568	54532	<0.001	5.025e-03	5.628e-03

Table S4.2. Number of tracks whose movement correlates significantly with footprint concentration (numbers to fig S4.3 e-f).
8.33% of tracks are straighter on FP
41.67% of tracks are straighter off FP
11.11% of tracks are faster on FP
30.56% of tracks are faster off FP

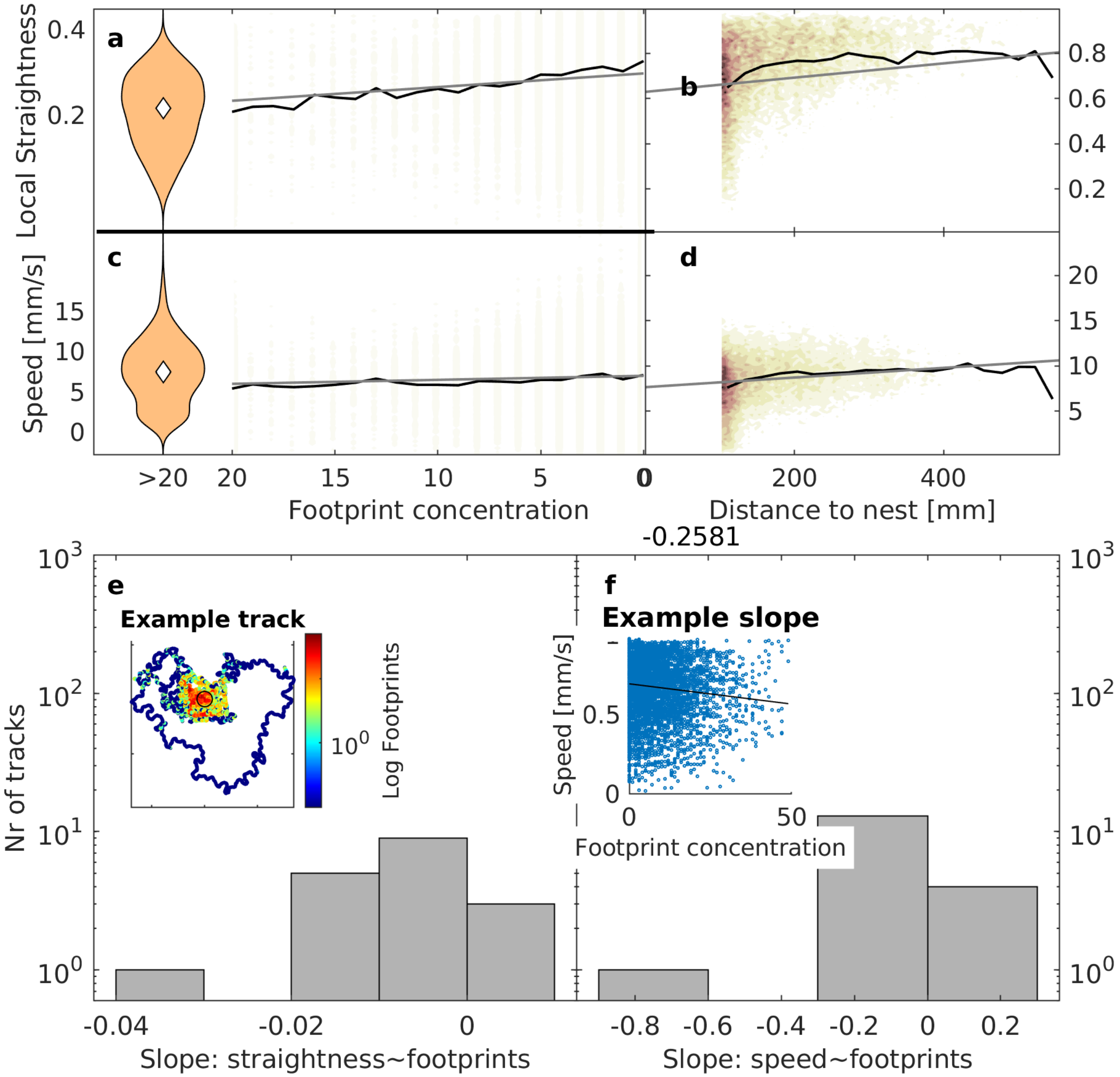

Fig S4.3. *Hunt et al. 2016 'NC' version of the main text figures 1 and 2. a), c) Straightness and speed increase with decreasing footprint concentration. b), d) Straightness and speed increases with distance to the nest. Black lines are medians per footprint and nest distance bins, respectively. Gray lines are regressions from the LMMs. Statistical tests were performed on unbinned data. Note that the x-axis of the footprint concentration panels are reversed to be consistent with the 'Distance to the nest' plots, as the highest footprint concentrations are around the nest (i.e. towards the left side on both graphs). The majority of ants walk e) straighter and f) faster on lower footprint concentrations (see tab S4.2 for numbers). Histograms are of the slopes (for each track) of the linear models of straightness or speed ~ footprint concentration. Negative values mean ants are walking less straight or slower with increasing footprint concentration. Left inset: example track, colored by the footprint concentration the ant is currently walking over. Black open circle indicates the nest location. Right inset: scatterplot with slope of the linear model of straight*ness~footprints for that track.

Table S4.4. No significant effect of straightness and speed at the start of the tracks with its mean distance (fig S4.5 e-f).

	**Estimate**	**SE**	**tStat**	**pValue**
st_slope	358.74	173.16	2.072	0.055
v_slope	8.87	5.221	1.699	0.109

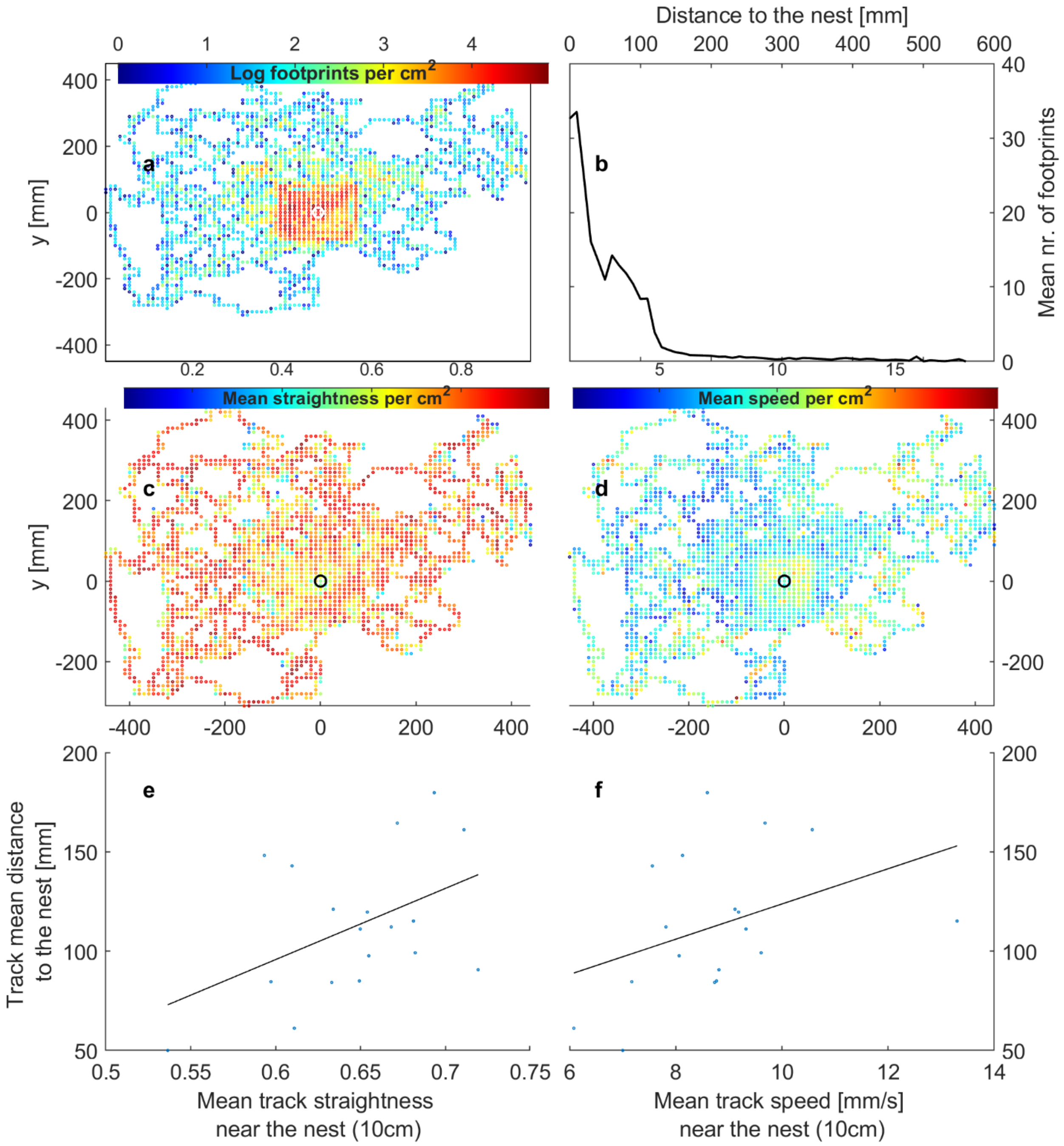

Fig S4.5. *Hunt et al. 2016 'NC' version of main text figures 3 and 4. a), c), d): Heatmaps of the last 5 min of the experiment. a) ant visitation, binned into pixels of size 1 cm. Points outside the black rectangle were omitted from analysis. Only data of the last hour of the experiment are included. Note that ants at different timepoints experience a slightly different footprint landscape. b) Mean footprint concentration by distance to the nest. The y-axis here is the x-axis in fig 1a, x-axis here is x-axis in fig 1b). This illustrates the mismatch between the x-axes in fig 1. c) & d) heatmaps of mean straightness and speed per pixel, respectively. In a), c) & d) white and black circles indicate nest locations. e) Tracks which start out straighter move on average farther away from the nest (where there are fewer footprints), but f) tracks which are faster near the nest move less far from the nest than those which are slower near the nest.*

Table S4.6. Stats to fig. S4.3 a: Lower straightness on higher footprint concentration irrespective of distance to the nest. pCorr stands for Benjamini-Hochberg corrected p-values. (fig S4.12 a)

Name	slope	SE	tStat	DF	pValue	Lower	Upper	pCorr
ST bin1	7.183E-04	1.008E-04	7.123	7207	<0.001	5.206E-04	9.160E-04	<0.001
ST bin2	-2.619E-04	1.362E-04	-1.922	7191	0.055	-5.289E-04	5.179E-06	0.068
ST bin3	3.521E-04	1.818E-04	1.936	7228	0.053	-4.329E-06	7.084E-04	0.068
ST bin4	3.142E-04	2.057E-04	1.527	7247	0.127	-8.906E-05	7.175E-04	0.149
ST bin5	-4.748E-04	2.198E-04	-2.160	7250	0.031	-9.057E-04	-4.394E-05	0.047
ST bin6	4.730E-04	2.413E-04	1.960	7254	0.050	4.976E-08	9.459E-04	0.068
ST bin7	-4.683E-04	1.745E-04	-2.683	7245	0.007	-8.105E-04	-1.261E-04	0.012
ST bin8	-6.473E-04	1.652E-04	-3.919	7250	0.000	-9.711E-04	-3.235E-04	<0.001
ST bin9	2.942E-04	2.013E-04	1.461	7243	0.144	-1.005E-04	6.888E-04	0.160
ST bin10	-8.659E-04	1.987E-04	-4.357	7251	<0.001	-1.255E-03	-4.764E-04	<0.001
ST bin11	-1.087E-03	2.038E-04	-5.334	7259	<0.001	-1.486E-03	-6.875E-04	<0.001
ST bin12	-1.949E-03	2.355E-04	-8.274	7257	<0.001	-2.410E-03	-1.487E-03	<0.001
ST bin13	-6.575E-04	2.395E-04	-2.745	7237	0.006	-1.127E-03	-1.880E-04	0.011
ST bin14	-4.089E-03	2.298E-04	-17.795	7253	<0.001	-4.539E-03	-3.638E-03	<0.001
ST bin15	-9.188E-04	8.118E-04	-1.132	7259	0.258	-2.510E-03	6.725E-04	0.271
ST bin16	-5.945E-03	1.159E-03	-5.129	7239	<0.001	-8.218E-03	-3.673E-03	<0.001
ST bin17	5.395E-04	1.507E-03	0.358	7259	0.720	-2.415E-03	3.495E-03	0.720
ST bin18	-8.545E-03	1.609E-03	-5.310	7259	<0.001	-1.170E-02	-5.390E-03	<0.001
ST bin19	-1.317E-02	2.098E-03	-6.277	7209	<0.001	-1.728E-02	-9.058E-03	<0.001
ST bin20	-2.410E-02	2.486E-03	-9.695	7209	<0.001	-2.897E-02	-1.923E-02	<0.001

Table S4.7. Stats to fig. S4.3 b: Mostly lower speed on higher footprint concentration across distances to the nest. pCorr stands for Benjamini-Hochberg corrected p-values. (fig S4.12 b)

Name	slope	SE	tStat	DF	pValue	Lower	Upper	pCorr
v bin1	3.672E-03	2.039E-03	1.801	7257	0.072	-3.255E-04	7.670E-03	0.090
v bin2	5.788E-03	2.886E-03	2.006	7252	0.045	1.313E-04	1.144E-02	0.060
v bin3	3.289E-02	3.711E-03	8.861	7255	<0.001	2.561E-02	4.016E-02	<0.001
v bin4	8.832E-03	4.343E-03	2.034	7259	0.042	3.188E-04	1.735E-02	0.060
v bin5	5.190E-02	4.462E-03	11.632	7258	<0.001	4.315E-02	6.064E-02	<0.001
v bin6	5.612E-02	5.010E-03	11.203	7259	<0.001	4.630E-02	6.594E-02	<0.001
v bin7	2.842E-02	3.746E-03	7.588	7258	<0.001	2.108E-02	3.577E-02	<0.001
v bin8	3.032E-02	3.692E-03	8.211	7258	<0.001	2.308E-02	3.755E-02	<0.001
v bin9	5.304E-02	4.001E-03	13.258	7258	<0.001	4.520E-02	6.088E-02	<0.001
v bin10	5.535E-02	3.944E-03	14.033	7259	<0.001	4.762E-02	6.308E-02	<0.001
v bin11	5.236E-02	3.658E-03	14.312	7260	<0.001	4.519E-02	5.953E-02	<0.001
v bin12	2.611E-02	4.100E-03	6.367	7259	<0.001	1.807E-02	3.414E-02	<0.001

v bin13	1.594E-02	4.190E-03	3.804	7259	<0.001	7.726E-03	2.415E-02	<0.001
v bin14	-5.624E-03	4.029E-03	-1.396	7259	0.163	-1.352E-02	2.273E-03	0.186
v bin15	4.633E-02	1.526E-02	3.035	7259	0.002	1.641E-02	7.625E-02	0.004
v bin16	3.198E-02	2.318E-02	1.380	7259	0.168	-1.346E-02	7.742E-02	0.186
v bin17	2.076E-01	3.150E-02	6.590	7259	<0.001	1.458E-01	2.693E-01	<0.001
v bin18	3.867E-02	3.352E-02	1.154	7259	0.249	-2.704E-02	1.044E-01	0.262
v bin19	-2.084E-02	4.275E-02	-0.487	7259	0.626	-1.046E-01	6.296E-02	0.626
v bin20	-1.244E-01	5.307E-02	-2.345	7259	0.019	-2.285E-01	-2.041E-02	0.029

Table S4.8. Spots where ants move less straight to begin with will accumulate more footprints (stats to fig S4.12c). Supplementary: LMM of maximum footprint number ~ initial straightness, with 'nest distance' and 'colony' as random factors: Estimate = -9.92, SE = 0.18, t = -54.79, DF = 1.45e6, p <0.001, Lower = -10.27, Upper = -9.56.

Nest dist.	slope	SE	tStat	DF	pValue	Lower	Upper	pCorr
bin 01	4.06	0.99	4.11	7104	<0.001	2.12	6.00	<0.001
bin 02	-5.61	0.93	-6.03	7092	<0.001	-7.44	-3.79	<0.001
bin 03	-9.40	0.78	-12.06	7129	<0.001	-10.93	-7.87	<0.001
bin 04	-6.34	0.78	-8.17	7250	<0.001	-7.87	-4.82	<0.001
bin 05	-9.76	0.73	-13.36	7253	<0.001	-11.19	-8.33	<0.001
bin 06	-8.24	0.68	-12.05	7258	<0.001	-9.58	-6.90	<0.001
bin 07	-16.78	0.91	-18.34	7259	<0.001	-18.58	-14.99	<0.001
bin 08	-20.29	1.01	-20.04	7242	<0.001	-22.28	-18.31	<0.001
bin 09	1.52	0.99	1.54	7230	0.124	-0.42	3.45	0.124
bin 10	-2.60	0.98	-2.66	7259	0.008	-4.52	-0.69	0.008
bin 11	-9.39	0.93	-10.08	7260	<0.001	-11.22	-7.57	<0.001
bin 12	3.51	0.82	4.26	7257	<0.001	1.89	5.12	<0.001
bin 13	-8.99	0.81	-11.16	7249	<0.001	-10.57	-7.41	<0.001
bin 14	-23.42	0.85	-27.53	7259	<0.001	-25.09	-21.75	<0.001
bin 15	-3.38	0.22	-15.34	7259	<0.001	-3.81	-2.94	<0.001
bin 16	-2.51	0.16	-16.17	7237	<0.001	-2.82	-2.21	<0.001
bin 17	-0.91	0.11	-8.29	7259	<0.001	-1.12	-0.69	<0.001
bin 18	-0.83	0.11	-7.53	7259	<0.001	-1.05	-0.62	<0.001
bin 19	-1.14	0.08	-13.95	7208	<0.001	-1.30	-0.98	<0.001
bin 20	-0.81	0.07	-11.50	7214	<0.001	-0.94	-0.67	<0.001

Table S4.9. Spots where ants move slower to begin with will accumulate more footprints (stats to fig S4.12d). Supplementary: LMM maximum footprint number ~ initial speed, with 'nest distance' and 'colony' as random factors: Estimate = 0.06, SE = 9.10e3, t = 6.37, DF = 1.45e6, p<0.001, Lower = 0.04, Upper = 0.08.

Nest dist.	slope	SE	tStat	DF	pValue	Lower	Upper	pCorr
bin 01	-2.00E-01	5.10E-02	-3.93	7260	<0.001	-3.00E-01	-1.01E-01	<0.001

bin 02	1.35E-01	4.94E-02	2.73	7240	0.006	3.78E-02	2.31E-01	0.008
bin 03	-1.62E-02	4.68E-02	-0.35	7247	0.729	-1.08E-01	7.55E-02	0.729
bin 04	-3.13E-01	3.92E-02	-7.98	7257	<0.001	-3.90E-01	-2.36E-01	<0.001
bin 05	-3.93E-01	4.11E-02	-9.56	7259	<0.001	-4.74E-01	-3.12E-01	<0.001
bin 06	-2.98E-01	3.82E-02	-7.79	7259	<0.001	-3.72E-01	-2.23E-01	<0.001
bin 07	-7.68E-01	5.09E-02	-15.09	7259	<0.001	-8.68E-01	-6.68E-01	<0.001
bin 08	-8.03E-01	5.18E-02	-15.50	7259	<0.001	-9.05E-01	-7.01E-01	<0.001
bin 09	-2.98E-01	5.06E-02	-5.88	7256	<0.001	-3.97E-01	-1.99E-01	<0.001
bin 10	1.71E-01	5.14E-02	3.33	7259	<0.001	7.03E-02	2.72E-01	0.001
bin 11	4.30E-01	5.67E-02	7.58	7260	<0.001	3.19E-01	5.41E-01	<0.001
bin 12	9.65E-02	4.85E-02	1.99	7259	0.047	1.42E-03	1.92E-01	0.049
bin 13	-5.53E-01	4.99E-02	-11.09	7259	<0.001	-6.51E-01	-4.56E-01	<0.001
bin 14	-1.12E+00	5.34E-02	-20.96	7259	<0.001	-1.22E+00	-1.01E+00	<0.001
bin 15	-5.22E-02	1.21E-02	-4.33	7259	<0.001	-7.59E-02	-2.86E-02	<0.001
bin 16	-3.41E-02	7.91E-03	-4.31	7259	<0.001	-4.96E-02	-1.86E-02	<0.001
bin 17	-2.19E-02	5.38E-03	-4.07	7259	<0.001	-3.24E-02	-1.13E-02	<0.001
bin 18	-3.32E-02	5.36E-03	-6.19	7259	<0.001	-4.37E-02	-2.27E-02	<0.001
bin 19	-9.92E-03	4.04E-03	-2.46	7259	0.014	-1.78E-02	-2.01E-03	0.016
bin 20	-2.33E-02	3.24E-03	-7.21	7259	<0.001	-2.97E-02	-1.70E-02	<0.001

Table S4.10. When the first point of a pixel was less straight than 0.5, ants walk straighter with increasing number of footprints, and when the first point of a pixel was straighter than 0.5, ants walk less straight with increasing number of footprints (stats to fig S4.12e)

st_bin	Estimate	SE	tStat	n	pValue
>= 0	5.80E-03	1.67E-04	34.71	7208	<0.001
>=0.27	4.71E-03	1.65E-04	28.47	7216	<0.001
>=0.37	3.49E-03	1.55E-04	22.58	7211	<0.001
>=0.44	1.95E-03	1.41E-04	13.90	7213	<0.001
>= 0.5	1.06E-03	1.29E-04	8.21	7216	<0.001
>=0.54	-1.30E-04	1.18E-04	-1.10	7217	0.270
>=0.58	-6.68E-04	1.22E-04	-5.47	7215	<0.001
>=0.61	-8.29E-04	1.35E-04	-6.15	7219	<0.001
>=0.64	-1.21E-03	1.34E-04	-9.04	7219	<0.001
>=0.67	-2.07E-03	1.31E-04	-15.84	7219	<0.001
>= 0.7	-1.92E-03	1.26E-04	-15.25	7217	<0.001
>=0.72	-2.74E-03	1.30E-04	-21.04	7225	<0.001
>=0.75	-3.34E-03	1.31E-04	-25.41	7220	<0.001
>=0.77	-4.03E-03	1.46E-04	-27.64	7225	<0.001
>=0.79	-4.63E-03	1.59E-04	-29.16	7224	<0.001
>=0.82	-5.07E-03	1.49E-04	-33.98	7223	<0.001
>=0.84	-5.70E-03	1.45E-04	-39.27	7224	<0.001
>=0.87	-6.22E-03	1.59E-04	-39.25	7224	<0.001

>=0.89	-7.42E-03	1.66E-04	-44.66	7224	<0.001
>=0.93	-9.79E-03	2.05E-04	-47.89	7225	<0.001

Table S4.11. When the first point of a pixel was slower than 9.3 mm/s, ants walk faster with increasing number of footprints, and when the first point of a pixel was slower than 9.3 mm/s, ants walk slower with increasing number of footprints (stats to fig S4.12f)

v_bin	Estimate	SE	tStat	n	pValue
>= 0.06	1.12E-01	3.09E-03	36.37	7259	<0.001
>= 2.38	7.82E-02	2.93E-03	26.69	7259	<0.001
>= 3.48	7.61E-02	2.91E-03	26.17	7259	<0.001
>= 4.41	6.67E-02	2.70E-03	24.71	7258	<0.001
>= 5.2	5.50E-02	2.61E-03	21.08	7258	<0.001
>= 5.86	4.29E-02	2.73E-03	15.70	7258	<0.001
>= 6.45	3.43E-02	2.63E-03	13.05	7260	<0.001
>= 6.97	3.15E-02	2.70E-03	11.65	7257	<0.001
>= 7.45	3.43E-02	3.01E-03	11.38	7258	<0.001
>= 7.91	1.38E-02	2.57E-03	5.35	7259	<0.001
>= 8.39	2.02E-02	2.81E-03	7.20	7260	<0.001
>= 8.82	1.17E-03	2.76E-03	0.42	7259	0.672
>= 9.28	4.72E-03	2.98E-03	1.58	7259	0.114
>= 9.73	-8.30E-03	2.86E-03	-2.90	7260	0.004
>=10.21	-2.68E-02	2.79E-03	-9.59	7259	<0.001
>=10.76	-2.39E-02	2.90E-03	-8.25	7258	<0.001
>=11.36	-4.32E-02	2.88E-03	-15.00	7258	<0.001
>=12.06	-5.99E-02	3.12E-03	-19.18	7257	<0.001
>=12.99	-1.00E-01	3.46E-03	-28.94	7258	<0.001
>=14.52	-1.64E-01	3.84E-03	-42.79	7259	<0.001

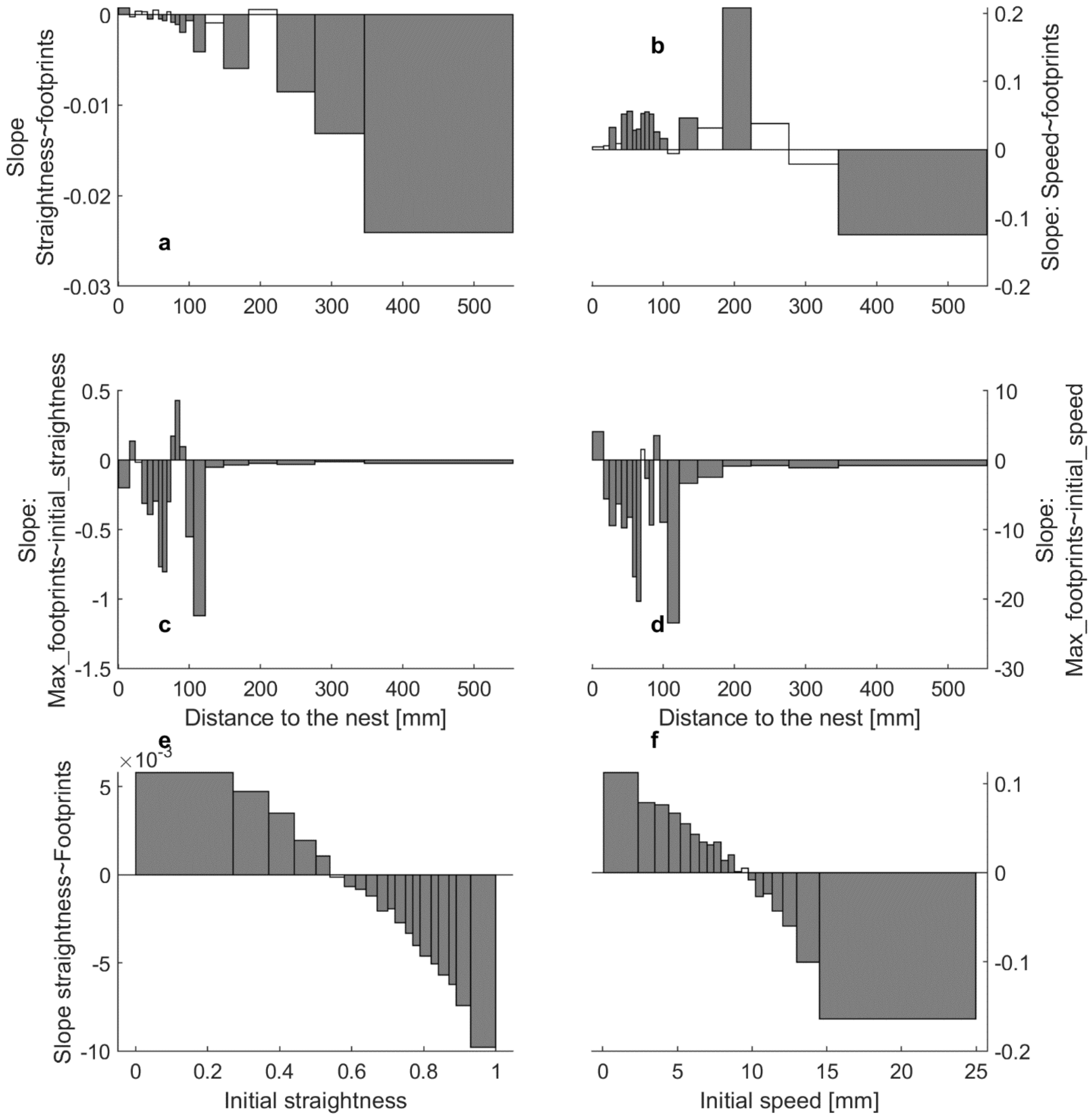

fig S4.12*. Hunt et al. 2016 'NC' version of main text figures 5, 6, and 7. Ants walk a) straighter and b) mostly faster with lower footprint concentration, even when binning by (= controlling for) distance to the nest. Bins are sized to each contain about 9.04x10^4 points. c) Slopes of mean final footprint number of 2x2 mm pixels over the straightness value of the first point created in that pixel, for each nest distance bin. d) Same as c), but with speed on the x-axis. e) slopes of the linear model of (straightness~footprints) over the straightness of the first point in the respective pixel. Negative values indicate ants walking straighter on lower footprint concentrations. f) same as e) for initial speed. Filled bars indicate values significantly different from 0.*

Ants do not turn preferentially in the direction of higher or lower footprint concentrations (Fig S4.13; LM: turn_angle ~ L-R, Estimate = -0.05, SE = 0.039, t = -1.31, n = 93008, p = 0.192].

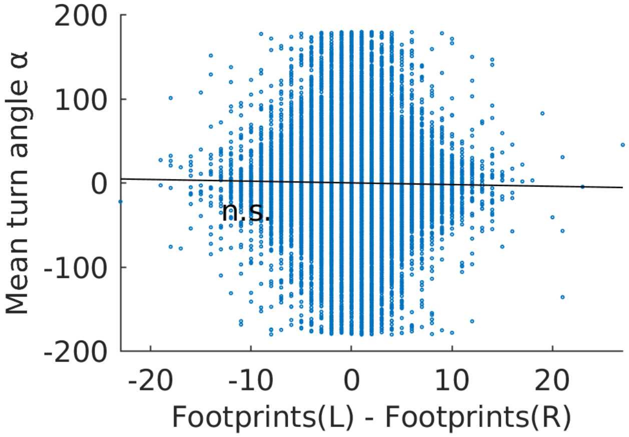

Fig. S4.13*. Hunt et al. 2016 'NC' version of main text figure 8. Ants do not turn toward or away from a footprint gradient. A negative slope would indicate turning toward the side of higher footprint concentrations.*
