# Supplementary material for: Collective search in ants: Movement determines footprints, and footprints influence movement
Source: PLoS One. 2024 Apr 23;19(4):e0299432. doi: 10.1371/journal.pone.0299432 (PMC11037541; doi:10.1371/journal.pone.0299432)
Supplement: S5 File — All main-text analyses on the ‘Cleaning’ data of Hunt et al. 2016. (PDF) [file pone.0299432.s005.pdf]

S5: Hunt et al. ‘C’ (no footprints between ants)

Table S5.1. Ants move straighter and slower further from the nest (fig S5.3 a-d). LMMs with ‘colony’ as a random factor.

Name	Estimate	SE	tStat	DF	pValue	Lower	Upper
st~c	-4.801e-03	1.972e-04	-43.329	54532	<0.001	-5.193e-03	-4.419e-03
v~c	-0.08467	3.706e-03	-16.038	54532	<0.001	-0.0919	-0.0774
st~nestDisp	1.18e-04	8.101e-06	39.803	54532	<0.001	1.021e-04	1.338e-04
v~nestDisp	5.692e-03	1.501e-04	34.568	54532	<0.001	5.398e-03	5.986e-03

Table S5.2. Number of tracks whose movement correlates significantly with footprint concentration (numbers to fig S5.3 e-f).

0% of tracks are straighter on FP

50% of tracks are straighter off FP

11.11% of tracks are faster on FP

30.56% of tracks are faster off FP

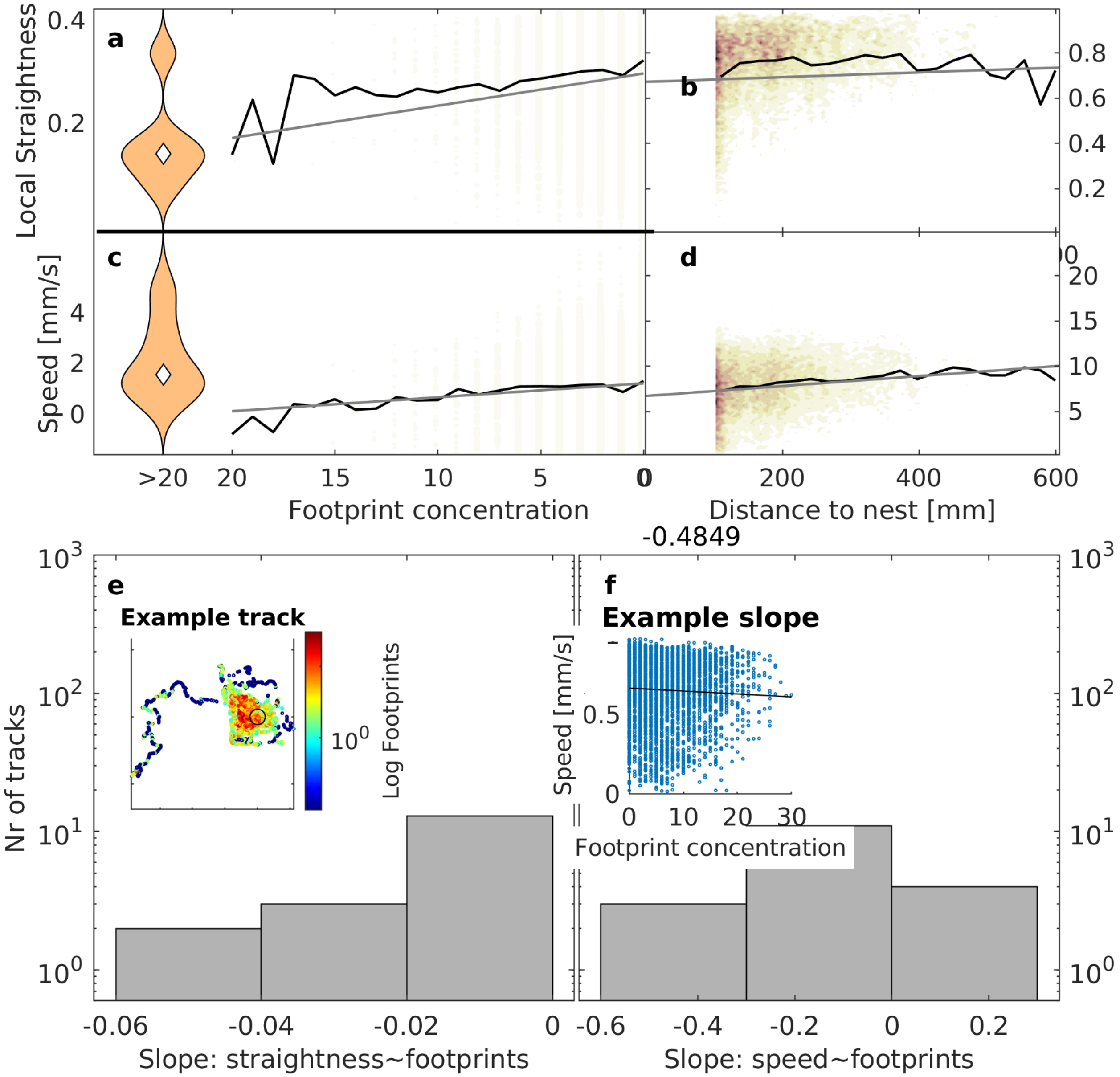

***Fig S5.3**. Hunt et al. 2016 'C' version of the main text figures 1 and 2. a), c) Straightness and speed increase with decreasing footprint concentration. b), d) Straightness and speed increases with distance to the nest. Black lines are medians per footprint and nest distance bins, respectively. Gray lines are regressions from the LMMs. Statistical tests were performed on unbinned data. Note that the x-axis of the footprint concentration panels are reversed to be consistent with the 'Distance to the nest' plots, as the highest footprint concentrations are around the nest (i.e. towards the left side on both graphs). More ants walk significantly e) straighter and f) faster on lower footprint concentrations than the opposite (see tab S5.2 for numbers). Histograms are of the slopes (for each track) of the linear models of straightness or speed ~ footprint concentration. Negative values mean ants are walking less straight or slower with increasing footprint concentration. Left inset: example track, colored by the footprint concentration the ant is currently walking over. Black open circle indicates the nest location. Right inset: scatterplot with slope of the linear model of straightness~footprints for that track.*

Table S5.4. No significant effect of straightness and speed at the start of the tracks with its mean distance (fig S5.5 e-f).

	Estimate	SE	tStat	pValue
st_intrcpt	13.426	192.54	0.06973	0.945
st_slope	150.65	286.22	0.5263	0.606
v_intrcpt	39.795	59.378	0.6702	0.512
v_slope	8.5683	6.6961	1.2796	0.219

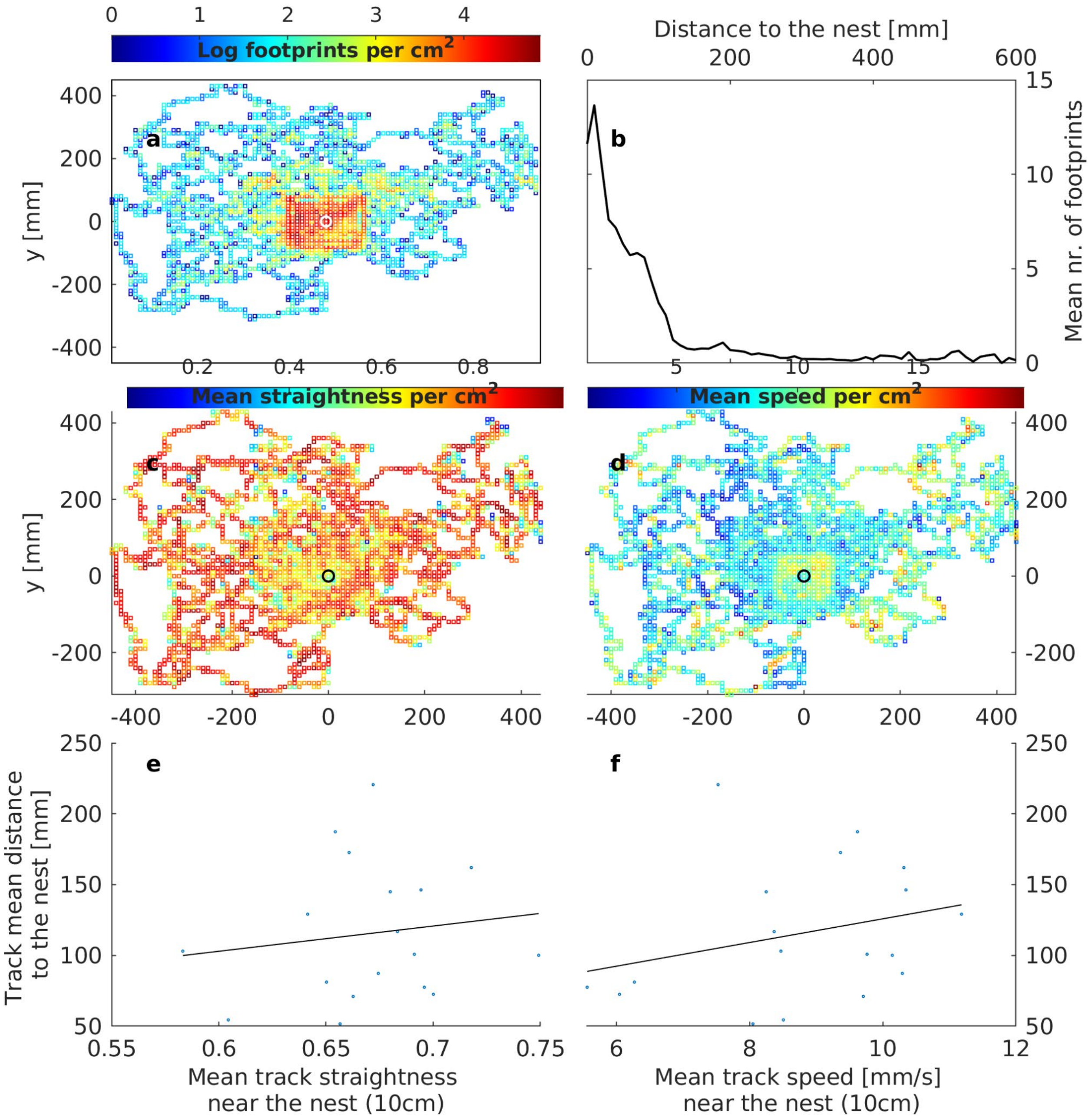

***Fig S5.5**. Hunt et al. 2016 ‘NC’ version of main text figures 3 and 4. a), c), d): Heatmaps of the last 5 min of the experiment. a) ant visitation, binned into pixels of size 1 cm. Points outside the black rectangle*

were omitted from analysis. Only data of the last hour of the experiment are included. Note that ants at different timepoints experience a slightly different footprint landscape. b) Mean footprint concentration by distance to the nest. The y-axis here is the x-axis in fig 1a, x-axis here is x-axis in fig 1b). This illustrates the mismatch between the x-axes in fig 1. c) & d) heatmaps of mean straightness and speed per pixel, respectively. In a), c) & d) white and black circles indicate nest locations. e) Tracks which start out straighter move on average farther away from the nest (where there are fewer footprints), but f) tracks which are faster near the nest move less far from the nest than those which are slower near the nest.

Table S5.6. Stats to fig. S5.3 a: Lower straightness on higher footprint concentration irrespective of distance to the nest. pCorr stands for Benjamini-Hochberg corrected p-values. (fig S5.12 a)

Name	slope	SE	tStat	DF	pValue	Lower	Upper	pCorr
ST bin1	2.246E-04	2.556E-04	0.879	6175	0.380	-2.764E-04	7.256E-04	0.447
ST bin2	-1.954E-03	3.890E-04	-5.022	6163	<0.001	-2.716E-03	-1.191E-03	<0.001
ST bin3	-1.573E-04	4.721E-04	-0.333	6190	0.739	-1.083E-03	7.682E-04	0.821
ST bin4	-6.974E-04	4.458E-04	-1.564	6217	0.118	-1.571E-03	1.765E-04	0.147
ST bin5	-2.229E-03	4.427E-04	-5.034	6216	<0.001	-3.096E-03	-1.361E-03	<0.001
ST bin6	-2.826E-03	4.658E-04	-6.066	6200	<0.001	-3.739E-03	-1.913E-03	<0.001
ST bin7	-3.735E-03	5.388E-04	-6.933	6219	<0.001	-4.791E-03	-2.679E-03	<0.001
ST bin8	1.077E-04	5.784E-04	0.186	6217	0.852	-1.026E-03	1.242E-03	0.897
ST bin9	8.650E-07	5.475E-04	0.002	6212	0.999	-1.072E-03	1.074E-03	0.999
ST bin10	-1.504E-03	5.357E-04	-2.807	6214	0.005	-2.554E-03	-4.537E-04	0.007
ST bin11	-5.258E-03	5.432E-04	-9.680	6212	<0.001	-6.323E-03	-4.194E-03	<0.001
ST bin12	-3.538E-03	5.940E-04	-5.956	6222	<0.001	-4.702E-03	-2.373E-03	<0.001
ST bin13	-3.559E-03	7.618E-04	-4.672	6222	<0.001	-5.052E-03	-2.066E-03	<0.001
ST bin14	-1.068E-02	9.749E-04	-10.960	6202	<0.001	-1.260E-02	-8.774E-03	<0.001
ST bin15	-6.541E-03	1.667E-03	-3.923	6205	<0.001	-9.810E-03	-3.272E-03	<0.001
ST bin16	-8.573E-03	1.333E-03	-6.429	6194	<0.001	-1.119E-02	-5.959E-03	<0.001
ST bin17	-9.750E-03	1.841E-03	-5.295	6179	<0.001	-1.336E-02	-6.141E-03	<0.001
ST bin18	-1.762E-02	2.367E-03	-7.446	6210	<0.001	-2.226E-02	-1.298E-02	<0.001
ST bin19	-2.635E-02	3.124E-03	-8.436	6212	<0.001	-3.248E-02	-2.023E-02	<0.001
ST bin20	-4.585E-02	2.871E-03	-15.972	6201	<0.001	-5.148E-02	-4.023E-02	<0.001

Table S5.7. Stats to fig. S5.3 b: Mostly lower speed on higher footprint concentration across distances to the nest. pCorr stands for Benjamini-Hochberg corrected p-values. (fig S5.12 b)

Name	slope	SE	tStat	DF	pValue	Lower	Upper	pCorr
v bin1	3.388E-02	5.501E-03	6.159	6220	<0.001	2.310E-02	4.466E-02	<0.001
v bin2	1.319E-02	8.370E-03	1.576	6216	0.115	-3.214E-03	2.960E-02	0.121
v bin3	5.649E-02	1.028E-02	5.497	6218	<0.001	3.635E-02	7.664E-02	<0.001
v bin4	5.955E-02	1.004E-02	5.929	6222	<0.001	3.986E-02	7.924E-02	<0.001
v bin5	3.099E-02	9.796E-03	3.164	6222	0.002	1.179E-02	5.020E-02	0.002
v bin6	3.615E-02	1.029E-02	3.514	6221	<0.001	1.598E-02	5.632E-02	0.001
v bin7	7.021E-02	1.176E-02	5.969	6221	<0.001	4.715E-02	9.327E-02	<0.001
v bin8	8.096E-02	1.219E-02	6.643	6221	<0.001	5.707E-02	1.049E-01	<0.001
v bin9	1.015E-01	1.153E-02	8.810	6222	<0.001	7.895E-02	1.241E-01	<0.001
v bin10	1.166E-01	1.019E-02	11.442	6220	<0.001	9.666E-02	1.366E-01	<0.001
v bin11	1.054E-01	9.308E-03	11.325	6222	<0.001	8.717E-02	1.237E-01	<0.001
v bin12	7.781E-02	9.842E-03	7.906	6222	<0.001	5.852E-02	9.710E-02	<0.001
v bin13	5.302E-02	1.310E-02	4.046	6222	<0.001	2.733E-02	7.871E-02	<0.001
v bin14	-7.789E-02	1.707E-02	-4.562	6222	<0.001	-1.114E-01	-4.442E-02	<0.001
v bin15	1.011E-01	3.065E-02	3.297	6222	0.001	4.098E-02	1.611E-01	0.001
v bin16	7.774E-02	2.530E-02	3.072	6222	0.002	2.814E-02	1.273E-01	0.003
v bin17	-9.122E-02	3.328E-02	-2.741	6222	0.006	-1.565E-01	-2.598E-02	0.007
v bin18	-2.284E-02	4.548E-02	-0.502	6222	0.616	-1.120E-01	6.632E-02	0.616
v bin19	-3.474E-01	6.422E-02	-5.410	6222	<0.001	-4.733E-01	-2.215E-01	<0.001
v bin20	-5.067E-01	5.226E-02	-9.696	6221	<0.001	-6.091E-01	-4.042E-01	<0.001

Table S5.8. Spots where ants move less straight to begin with will accumulate more footprints (stats to fig S5.12c). Supplementary: LMM of maximum footprint number ~ initial straightness, with 'nest distance' and 'colony' as random factors: Estimate = -3.13, SE = 0.08, t = -39.84, DF = 1.24e5, p<0.001, Lower = -3.29, Upper = -2.98.

Nest dist.	slope	SE	tStat	DF	pValue	Lower	Upper	pCorr
bin 01	-1.46	0.58	-2.54	6090	0.011	-2.59	-0.34	0.011
bin 02	-2.63	0.51	-5.18	6154	<0.001	-3.63	-1.64	<0.001
bin 03	1.95	0.39	5.03	6094	<0.001	1.19	2.71	<0.001
bin 04	-3.17	0.40	-7.86	6221	<0.001	-3.96	-2.38	<0.001
bin 05	-6.84	0.43	-15.80	6221	<0.001	-7.69	-5.99	<0.001
bin 06	-7.30	0.42	-17.52	6204	<0.001	-8.12	-6.49	<0.001
bin 07	-1.10	0.40	-2.76	6221	0.006	-1.87	-0.32	0.006
bin 08	-1.26	0.35	-3.62	6214	<0.001	-1.95	-0.58	<0.001
bin 09	-2.30	0.35	-6.62	6201	<0.001	-2.98	-1.62	<0.001
bin 10	-3.91	0.37	-10.45	6221	<0.001	-4.65	-3.18	<0.001
bin 11	-8.43	0.38	-22.35	6203	<0.001	-9.17	-7.69	<0.001
bin 12	-7.16	0.36	-19.86	6222	<0.001	-7.87	-6.45	<0.001
bin 13	-3.23	0.28	-11.38	6222	<0.001	-3.79	-2.68	<0.001
bin 14	-3.94	0.22	-17.57	6193	<0.001	-4.38	-3.50	<0.001
bin 15	-0.63	0.12	-5.27	6210	<0.001	-0.86	-0.39	<0.001
bin 16	-2.32	0.15	-15.00	6190	<0.001	-2.62	-2.02	<0.001

bin 17	-1.41	0.11	-13.13	6184	<0.001	-1.62	-1.20	<0.001
bin 18	-1.05	0.08	-12.61	6210	<0.001	-1.21	-0.89	<0.001
bin 19	-0.40	0.06	-6.38	6212	<0.001	-0.53	-0.28	<0.001
bin 20	-1.85	0.07	-26.51	6206	<0.001	-1.99	-1.71	<0.001

Table S5.9. Spots where ants move slower to begin with will accumulate more footprints (stats to fig S5.12d). Supplementary: LMM of maximum footprint number ~ initial speed, with 'nest distance' and 'colony' as random factors: Estimate = 0.12, SE = 3.88e3, t = 31.71, DF = 1.24e5, p>0.001, Lower = 0.12, Upper = 0.13.

Nest dist.	slope	SE	tStat	DF	pValue	Lower	Upper	pCorr
bin 01	-9.71E-02	3.12E-02	-3.11	6204	0.002	-1.58E-01	-3.60E-02	0.002
bin 02	-2.56E-01	2.37E-02	-10.79	6210	<0.001	-3.02E-01	-2.09E-01	<0.001
bin 03	-1.87E-02	1.71E-02	-1.09	6208	0.276	-5.23E-02	1.49E-02	0.307
bin 04	-2.26E-01	1.86E-02	-12.17	6222	<0.001	-2.63E-01	-1.90E-01	<0.001
bin 05	-1.56E-01	2.13E-02	-7.31	6222	<0.001	-1.98E-01	-1.14E-01	<0.001
bin 06	-1.18E-01	2.00E-02	-5.89	6218	<0.001	-1.57E-01	-7.85E-02	<0.001
bin 07	-6.16E-02	1.73E-02	-3.56	6221	<0.001	-9.54E-02	-2.77E-02	<0.001
bin 08	3.18E-02	1.62E-02	1.96	6222	0.05	1.66E-05	6.36E-02	0.059
bin 09	1.16E-01	1.79E-02	6.50	6222	<0.001	8.11E-02	1.51E-01	<0.001
bin 10	1.05E-01	2.06E-02	5.08	6221	<0.001	6.42E-02	1.45E-01	<0.001
bin 11	1.49E-01	2.40E-02	6.21	6222	<0.001	1.02E-01	1.96E-01	<0.001
bin 12	-8.97E-02	2.40E-02	-3.74	6222	<0.001	-1.37E-01	-4.27E-02	<0.001
bin 13	-1.70E-03	1.71E-02	-0.10	6222	0.921	-3.53E-02	3.19E-02	0.921
bin 14	-2.15E-01	1.33E-02	-16.15	6222	<0.001	-2.41E-01	-1.89E-01	<0.001
bin 15	-3.14E-02	6.29E-03	-4.99	6222	<0.001	-4.37E-02	-1.90E-02	<0.001
bin 16	-8.19E-03	8.55E-03	-0.96	6222	<0.001	-2.50E-02	8.57E-03	0.356
bin 17	-2.15E-02	6.07E-03	-3.55	6222	<0.001	-3.34E-02	-9.65E-03	<0.001
bin 18	-1.48E-02	4.44E-03	-3.33	6222	<0.001	-2.35E-02	-6.07E-03	0.001
bin 19	-7.78E-03	3.10E-03	-2.51	6222	0.012	-1.39E-02	-1.70E-03	0.015
bin 20	-4.53E-02	4.00E-03	-11.32	6221	<0.001	-5.31E-02	-3.74E-02	<0.001

Table S5.10. When the first point of a pixel was less straight than 0.6, ants walk straighter with increasing number of footprints, and when the first point of a pixel was straighter than 0.6, ants walk less straight with increasing number of footprints (stats to fig S5.12e)

st_bin	Estimate	SE	tStat	n	pValue
>= 0	2.02E-02	5.05E-04	39.96	6183	<0.001
>=0.28	1.47E-02	4.26E-04	34.36	6184	<0.001
>=0.37	8.90E-03	3.78E-04	23.58	6187	<0.001
>=0.45	6.06E-03	3.66E-04	16.55	6185	<0.001
>= 0.5	3.22E-03	3.34E-04	9.64	6184	<0.001
>=0.55	1.00E-03	2.86E-04	3.50	6189	<0.001

>=0.59	1.10E-03	3.34E-04	3.29	6190	0.001
>=0.62	-1.28E-03	3.18E-04	-4.02	6190	<0.001
>=0.66	-1.99E-03	3.37E-04	-5.90	6189	<0.001
>=0.68	-4.53E-03	3.33E-04	-13.60	6188	<0.001
>=0.71	-5.16E-03	3.47E-04	-14.84	6186	<0.001
>=0.74	-6.20E-03	2.99E-04	-20.72	6188	<0.001
>=0.76	-8.35E-03	3.59E-04	-23.26	6192	<0.001
>=0.79	-8.49E-03	3.05E-04	-27.79	6193	<0.001
>=0.81	-1.18E-02	3.74E-04	-31.65	6189	<0.001
>=0.83	-1.30E-02	3.71E-04	-35.07	6188	<0.001
>=0.85	-1.30E-02	3.56E-04	-36.47	6191	<0.001
>=0.88	-1.50E-02	3.66E-04	-40.93	6191	<0.001
>= 0.9	-1.89E-02	4.16E-04	-45.33	6193	<0.001
>=0.93	-2.07E-02	4.46E-04	-46.35	6190	<0.001

Table S5.11. When the first point of a pixel was slower than 9 mm/s, ants walk faster with increasing number of footprints, and when the first point of a pixel was slower than 9 mm/s, ants walk slower with increasing number of footprints (stats to fig S5.12f)

v_bin	Estimate	SE	tStat	n	pValue
>= 0.15	3.46E-01	8.34E-03	41.54	6222	<0.001
>= 1.97	2.54E-01	7.72E-03	32.84	6219	<0.001
>= 2.94	2.30E-01	6.92E-03	33.23	6222	<0.001
>= 3.84	2.10E-01	6.83E-03	30.75	6221	<0.001
>= 4.57	2.17E-01	7.20E-03	30.19	6221	<0.001
>= 5.22	2.21E-01	8.12E-03	27.23	6221	<0.001
>= 5.82	1.71E-01	6.98E-03	24.51	6222	<0.001
>= 6.33	1.41E-01	6.97E-03	20.21	6221	<0.001
>= 6.83	1.68E-01	7.35E-03	22.90	6222	<0.001
>= 7.33	1.06E-01	7.16E-03	14.74	6221	<0.001
>= 7.82	5.92E-02	6.60E-03	8.97	6219	<0.001
>= 8.29	6.95E-02	7.24E-03	9.60	6221	<0.001
>= 8.79	3.63E-02	7.55E-03	4.80	6221	<0.001
>= 9.33	-3.15E-04	7.14E-03	-0.04	6222	0.965
>= 9.9	-3.66E-02	7.30E-03	-5.01	6221	<0.001
>= 10.5	-8.00E-02	7.68E-03	-10.41	6222	<0.001
>=11.19	-1.13E-01	7.29E-03	-15.52	6219	<0.001
>=11.96	-1.79E-01	7.96E-03	-22.54	6221	<0.001
>=12.99	-2.34E-01	8.36E-03	-28.03	6221	<0.001
>=14.62	-4.27E-01	1.06E-02	-40.16	6221	<0.001

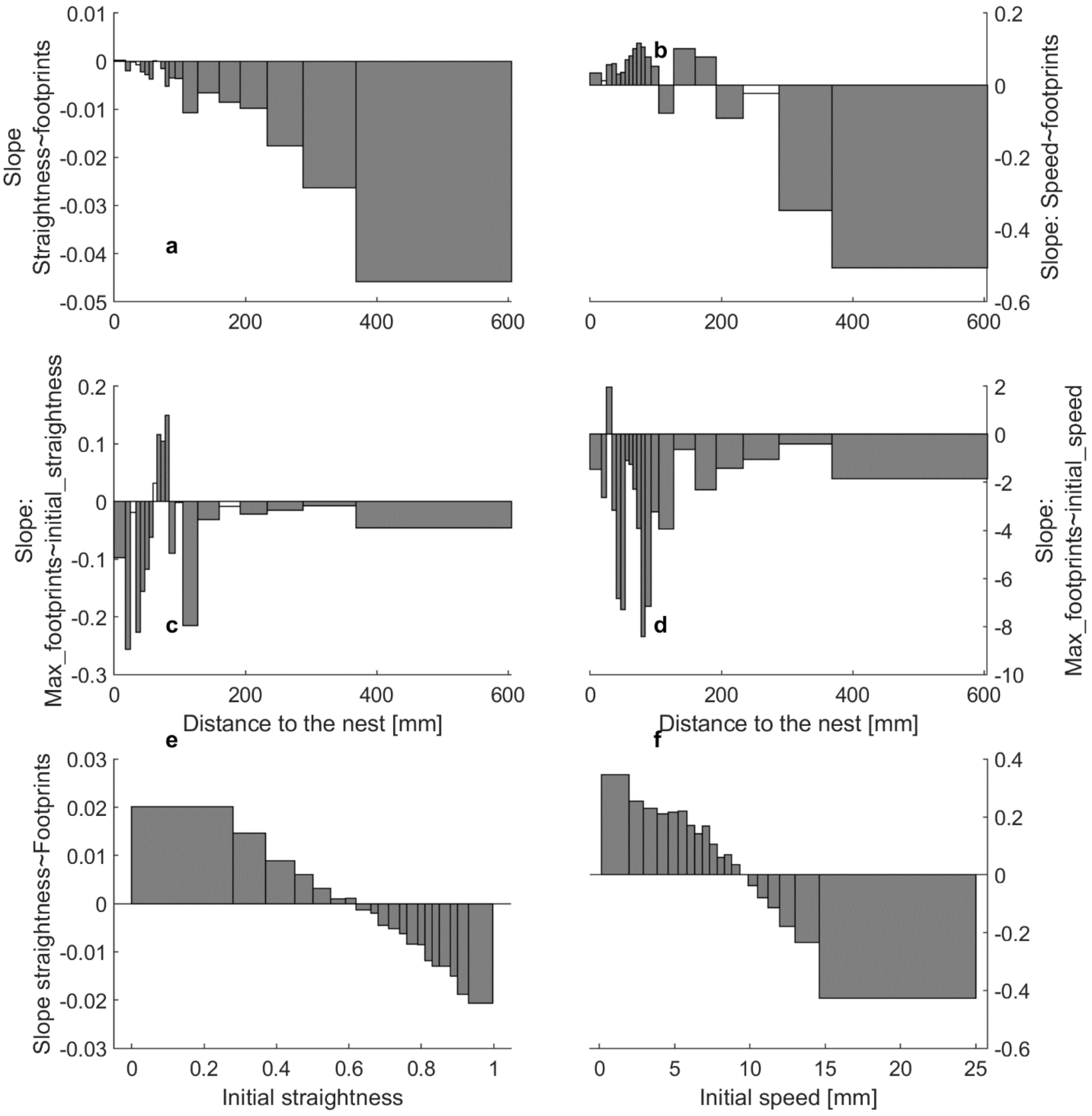

Fig. S5.12*. Hunt et al. 2016 'C' version of main text figures 5, 6, and 7. Ants walk a) straighter and b) mostly faster with lower footprint concentration, even when binning by (= controlling for) distance to the nest*. *Bins are sized to each contain about 9.04x10^4 points. c) Slopes of mean final footprint number of 2x2 mm pixels over the straightness value of the first point created in that pixel, for each nest distance bin. d) Same as c), but with speed on the x-axis. e) slopes of the linear model of (straightness~footprints) over the straightness of the first point in the respective pixel. Negative values indicate ants walking straighter on lower footprint concentrations. f) same as e) for initial speed. Filled bars indicate values significantly different from 0.*

Ants do not turn preferentially in the direction of higher or lower footprint concentrations (fig S5.13; LM: turn_angle ~ L-R, Estimate = -0.074, SE = 0.039, t = -1.894, n = 84033, p = 0.058).

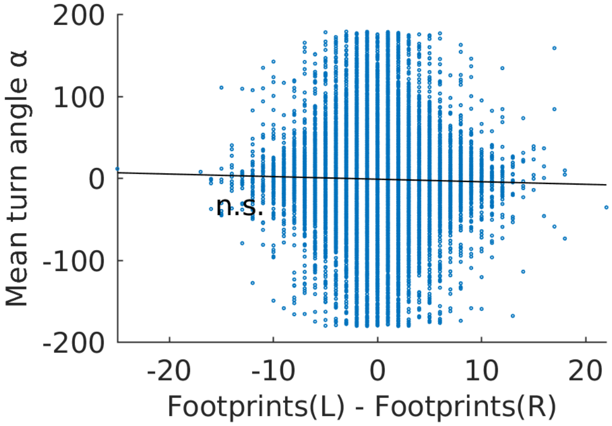

Fig. S5.13*. Hunt et al. 2016 'C' version of main text figure 8. Ants do not turn toward or away from a footprint gradient. A negative slope would indicate turning toward the side of higher footprint concentrations.*
