# Supplementary material for: Collective search in ants: Movement determines footprints, and footprints influence movement
Source: PLoS One. 2024 Apr 23;19(4):e0299432. doi: 10.1371/journal.pone.0299432 (PMC11037541; doi:10.1371/journal.pone.0299432)
Supplement: Attachment — Submitted filename: Response to review FP vs variation.pdf [file pone.0299432.s006.pdf]

Dear Editor,

Thank you for your positive evaluation of our work and we are happy to address your and the reviewer's comments.

Below are in blue our responses to the comments.

Unprompted changes:
We double-checked the cause of the small peak around 800 cm in Fig. 3b and removed 313 points which were false positive tracking errors and had not been removed before. Hence, we reran all analyses which contain these data points.
Fig. 6: We show in Fig. 5 that one should control for nest distance effects but do not take this into account in (the results of) Fig. 6. We thus changed Fig. 6 from line plots showing the correlations regardless of nest distance, to bar plots with correlations of points separated into nest distance bins, analogous to Fig. 5.
Heatmaps: We changed the appearance of the pixel in all heatmaps, since they had been displayed too big and thus overlapping.

Editor
I want to add an invitation to revise the Abstract. That section would benefit from 1-2 initial sentences placing the big picture topic that your project addresses and 1-2 sentences, in the end, going back to the main implications/novelty/significance of your results. This might result in not mentioning ants in those sentences, which will allow for a closer examination of the broad implications of your work. You do a good job with this in the Introduction and Conclusion. However, that needs to be better reflected in the abstract.
We changed the first few sentences of the abstract and added an implications sentence at the end.

Reviewer #1:

I found the paper very interesting, well-designed, and easy to read. Their major strength is that the authors present several ideas that test, guiding the reader to the reasons why reject or not reject the hypotheses discussed, and showing alternative explanations in each case. Their major weakness –but not really major- is the way in which the hypotheses are written, and some figures that I found little didactic if their function is to show patterns.
Below I describe these points more in detail hoping that these suggestions help the authors to make a more didactic and clear paper.
We thank you for your helpful comments and believe that our changes to the title and figure 1 made this a clearer manuscript.

The formulated hypotheses are in fact predictions. The real hypotheses should be the

mechanisms that generate these results (i.e., turning away from sites with higher footprint concentrations, or changing their turning patterns depending on the presence of footprints). Maybe the key hypothesis is that collective search is modulated by chemical footprints in order to maximize search cover, with the associated expected results regarding turning patterns.
We agree that the collective search modulation is the overarching (ultimate) hypothesis, but view klinotaxis and klinokinesis as two mechanistic sub-hypotheses, from which the predictions of turn angle bias and different straightnesses follow.

The title is a little confusing for me. What exactly means "…but indirect effects are large?"
For maximum clarity, we changed the title to "Searching ants do not avoid chemical footprints, but geometric artifacts have large effects and make causal effects of pheromone hard to detect

What is the number of replicates? I suppose, the number of ants analyzed?
We added a sentence in the General setup section with these numbers.

The authors refer to Fig. 1 to comment on their key result "ants moving straighter and faster when walking across fewer assumed footprints. For me, it is hard to see that pattern (i.e., a negative correlation between local straightness and footprint concentration) in the figure. The reverse x-axis does not help.
We zoomed in on the y-axis to better show the slope without cutting off too much important information. We chose to keep the x-axis reversed for better compatibility between the two sides of this figure.

Reviewer #2:

This study by Popp and Dornhaus presents a systematic investigation of searching movement behavior in the rock crevice ant, Temnothorax rugatulus. I find this research to be of interest for its contribution to our understanding of the effects of hypothetical chemical "footprints" on forager/searching behavior in T. rugatulus, although, the lack of a "clean" arena experiment and lack of chemical evidence somewhat limits the conclusions the authors can draw. The authors present interesting results regarding the correlation between nest distance, "footprint" concentration, and ant searching behaviors, i.e., path straightness and speed, which are important factors for maximizing efficient exploration of novel space. I think this study opens questions about what chemicals (e.g., cuticular hydrocarbons, volatile compounds, or both) might be contributing to the study's findings and how these potential chemicals interact with other factors (e.g., environmental cues, intrinsic behavioral biases, and other cues/signals from nestmates, etc.). In general, the authors do a fairly good job of summarizing their findings and discussing the implications of their work and its limitations. While many of my detailed comments in the manuscript relate to suggested edits or clarifications, several comments also relate to the analyses and conclusions drawn

(see questions/comments/suggestions summarized by line number below).
We thank you for your extremely thorough and helpful review. Your comments made us aware of an error in one of our analyses and they helped us describe our study more clearly and accurately. We traded off a ‘clean’ arena treatment with the large number of ants simultaneously tracked, as it would have been unfeasible to clean the arena after each exploration bout for hundreds or thousands of exploration bouts. Our approach lead us to detect the effects of interindividual variation and sensitivity to environmental cues, which we regard as perhaps the most important outcomes of this study.

Questions/comments/suggestions on the main manuscript:

Line 46: Change “…in addition…” to “…additionally…”
Done.

Line 55: Change “The authors of Hunt et al. speculate…” to “The research of Hunt et al. hypothesized…”
Done.

Line 68/69: Change “…plausible that also ants might use…” to “…plausible that ants might also use…”
Done also.

Line 102: Remove “…at least…”
Done.

Line 115: Change “…we filmed…” to “…we digitally video recorded…” (unless you actually video recorded them on film)
Since a) the commonly understood meaning of ‘to film’ has expanded from analogue to digital video recordings with the advent of the latter, and b) “digitally video recorded” unnecessarily complicates the sentence, we would like to stick with our initial phrasing.

Line 116: Add commas after “ants” and “wild,” or split the information into two sentences. It’s a bit clunky to read in its current form.
Split into two sentences.

Line 123: Change “…closer than ca. 5-20 cm to…” to “…less than 20 cm from…”
Done.

Line 153: Do you mean 40 mm (not 40 cm)?
Yes! Done.

Line 172: Do you mean Fig. 3a-b (not Fig. 2a-b)?
Done.

Line 177: Please indicate the specific panel of the figure for clarity, i.e., Fig. 6a.
Done.

Line 178: Consider adding “a” and “b” labels to the two panels of Fig. 7 and, again, indicate the specific panel of the figure for clarity, i.e., Fig. 7a.
Done.

Line 183: Change “…ant movements are straighter…” to “…ant movements significantly are straighter…” to indicate statistical support for the relationship.
Done.

Line 186: Do you mean Fig. S1 2a (not Fig. S1 3a)?
Yes! Done.

Lines 195-201: Please include a description of the faint vertical lines (i.e., heat maps) in panels a and c as well as the heat maps in panels b and d. I assume they are meant to represent individual data points, but these details are missing from the caption.
Done: These are both kernel density estimations of raw points.

Line 196: Please include units for footprint concentration, e.g., units per pixel, or whatever is the accurate measurement.
Done.

Line 201: Add a comma after “i.e.” (and elsewhere throughout the manuscript).
Done.

Lines 204-205 (re: Figure 2a): Maybe you could highlight the representative bars for the two highlighted categories using either different colors or a black/grey/white combo to make them easier for the reader to identify in the results.
Done:

Lines 206-208 (re: Figure 2b): Same comment as above.
Done.

Lines 229-235: Include somewhere in the Fig. 3 caption that this is representative of all 5 colonies since not everyone will look through the supplementary figures.
Done.

Lines 233-234: Remove “The y-axis here is the x-axis in fig 1a, x-axis here is x-axis in fig 1b. This illustrates the mismatch between the x-axes in fig 1.”, as this doesn’t seem particularly necessary to mention to understand the figure.
Done.

Line 248: Change “further” to “farther”.
Furthermore done.

Line 254: Change “…(where there are fewer footprints)…” to “…(where there are also fewer footprints, Fig. 3b)…”
Also done.

Line 267: The authors state, “…non-significant or positive between roughly 45 and 70 cm…”, but, according to the stats in table S1.5, bin 17 (roughly 1200-1300 mm) is also not significant.
Added.

Line 272: The caption states, “(only one bar is unfilled…”, but, again, bin 17 on Figure 5b should be unfilled as well.
Done.

Line 285: Please indicate the specific panel of the figure for clarity, i.e., Fig. 6a.
Done.

Lines 291-293: Since the speed vs. footprint relationship was not statistically relevant, Fig. 6b might be better in the supplementary materials.
We think that it benefits the reader by keeping all figures in the same format (i.e., straightness left and speed right), making it easier to identify the patterns.

Line 301: Change “Higher footprint concentrations cause ants to move less straight” to “Ants move less straight on higher footprint concentrations”. Claiming causality based on correlative results (even if there is a causal relationship) is inappropriate given the lack of experimental treatments in this study.
Done.

Line 309: The authors state, “…with bin widths of 0.1 straightness…”, but the bin widths appear to vary from less than 0.1 up to 0.5. Is this a typo or am I misinterpreting this? Also, the caption for Figure 7 reads, "...bars contain the same number of points (n = 88931) and are thus differently sized..." (Lines 320-321) so there seems to be contradictory info.
We changed that Results sentence to reflect that, in fact, bins contain the same number of

points. We clarified that the bin width in the figures are different, while containing the same number of points.

Line 310: Please indicate the specific panel of the figure for clarity, i.e., Fig. 7a.
Done.

Line 315: The summary of Figure 7b is completely missing here. While the results at lower initial speeds (<10 mm/s) largely match predictions, the results at the initial higher speeds (ca. 15-25 mm/s) have a significantly positive correlation between speed and footprints). This seems especially unusual given the prediction that more footprints lead to slower-moving ants and vice versa. What do you think is going on here? Is there a way to determine where these points cluster in space? Perhaps some uncontrolled factor is at play?
Extra thanks for bringing this up: When looking into this, we spotted a bug which resulted in the pixels for the analyses of Fig. 6 and 7 being much smaller than 2 mm. We thus updated those two figures (more on Fig. 6 at the end of this document) and the results text. In short, we see a pattern of steadily decreasing slopes with increasing initial straightness and speed, which can be explained with a ‘regression to the mean’ effect.

Line 339: This sentence reads a little abruptly. Consider adding something like, "The results of the turning direction analysis show that..." to the beginning of the statement.
Done.

Line 355: Change “short” to “intermediate”.
Done

Line 355: Do you mean Fig. S3.12b (not Fig. S3.5)?
Yes! Done.

Line 358: Do you mean excluded (instead of included)?
No, we mean to say that the correlation flips if nothing is excluded, and removed the unnecessary last part of the sentence.

Line 358: Please indicate the specific panel of the figure for clarity, i.e., Fig. S3.3d.
Done.

Line 364-365: Change “…to test whether our effects persist…” to “…to test whether the same effects persist…” since these are not data from your study.
Done.

Line 366: Add “methods” after analysis.
Done.

Lines 366-368: For clarity, please include a brief summary of the similarities/differences of the Hunt et al. studies/experiments to your study.
We added/changed this section: “While their general methods are similar to ours, there are a few key differences: they let 6 ants from each of their 3 colonies explore their arena one after another, and repeated this experiment while removing the chemical footprints after each ant’s trip (‘cleaning’, as opposed to ‘no cleaning’ in the other trials). They also only analyzed large-scale properties of the movement behavior (i.e., ant distributions) and walking speed, but not path straightness.”

Line 370: Please indicate the specific panels of the figure for clarity, i.e., Fig. S4.5e & f.
Done.

Line 372: Do you mean Fig. S4.12b (not Fig. S4.4b)?
Yes! Done.

Lines 374-375 and 377.5-381: Most of this is an interpretation of the results, which should go into the discussion or be removed if it is already present in the discussion.
We changed the first sentence in accordance with the changes in response to your next comment and removed the second sentence.

Lines 376-377.5: This can move to the beginning of the paragraph to help explain the context, i.e., see earlier comment for lines 366-368.
Done.

Line 386: It seems that the authors of reference 20 didn’t directly investigate the relationship between straightness/speed and footprint concentration, so this claim should be reworded to reflect the differences/similarities more accurately between your findings and this study.
We changed the sentence to reflect that the results of the cited studies lead to these hypotheses.

Line 389: Add “T. rugatulus” before “ants” for extra clarity.
Done.

Line 397: Change “…ants display klinokinesis…” to “…ants likely display klinokinesis…” since the conclusion is based on correlative results.
Done.

Line 400: Remove “also”.
Done.

Line 403: Change “warning” to “reminder”.
Done.

Line 404: Add a comma after “e.g.”.
Done.

Line 408: Change “Chemical footprints make ants walk less straight” to “Ants walk less straight on chemical footprints”. See earlier comment for line 301.
Done.

Line 411: Change “…previous study on another species in the same genus…” to “…a previous study on Temnothorax albipennis,...”.
Done.

Lines 411-412: The authors state, “…ants spend more time in unmarked areas”, but (unless I’m mistaken) the amount of time spent in each area versus footprint concentration was not measured in this study or your study, so is it correct to make this claim/comparison? In theory, couldn't ants walk faster and straighter on lower footprint concentrations and spend more total time in lower footprint concentrations?
This reference points to a study on *Temnothorax unifasciatus*, not one of the Hunt et al. papers. In that study, the authors analyzed time spent in different areas.

Line 416: Change”… seem to make ants walk less straight.” to “…correlate with ants walking less straight.”
Done.

Line 428: Change “instantaneously” to “quickly”.
Quickly done.

Line 431: Change “pheromone” to “pheromones”
Done.

Line 451: Add a comma after “e.g.”.
Done.

Line 454: Change “Spatial heterogeneity: Some areas make ants walk less straight…” to “Spatial heterogeneity: Ants walk less straight in some areas…”.
Done.

Line 486: Change “which is also called” to “i.e.,”.
Done.

Line 489: Change “…we do not find such behavior in our data…” to “…we do not find evidence for such behavior in the analysis of our data…”.
Done.

Line 494: Add “methods” after analysis.
Done.

Line 494: This second part of this sentence is difficult to follow, i.e., “…we found similar effects to our results within trials, except that here ants…”. Do you mean “we found similar effects in our results for within treatment analyses (i.e., "cleaning" [C] and "no cleaning" [NC] treatments), except that in our study ants…”? If not, please clarify.
We changed “within trials” to “in both of their treatments”.

Line 507: Change “…our experiments might be due to…” to “…our experiments, although not measured, might be due to…”.
Done.

Line 508: Change “private” to “individual”.
Done.

Line 512: Change “…according to our analysis (and our data support of these effects).” to “…according to our analysis methods, the results of which support of these effects.”.
Done.

Line 525: Do you mean 2 x 24 hours (not 2x5 hours)? The methods mentioned that day 3 recordings were used.
The ants were kept inside their nests (i.e. not allowed into the arena) for the time between the 5-h-long trials. They thus only had 2x5 h to familiarize themselves.

Lines 553-555: Change “We found no evidence for klinotaxis (turning towards or away from a cline of pheromone density). We found klinokinesis, i.e. an…” to “We found no evidence for klinotaxis (turning towards or away from a cline of pheromone density), but we did find evidence for klinokinesis, i.e., an…”
Done.

Lines 557-558: The authors state, “…ants seem to move in ways that make them spend more time in such areas.”, which is speculative, but the claim would have more power if the

study also included an analysis of the amount of time spent in an area vs. footprint concentration.
We changed the phrasing to “walk more distance”, since we are less interested in the time spent, than the area explored.

Line 560: Please be more specific about what is meant by “geometric correlations”.
We changed this to say “correlations expected from random walks (i.e., straighter movements tend to lead farther away from the origin” to indicate biologically non-interesting correlations.

Questions/comments/suggestions on the supplemental materials:

Figure S3.3 caption: The authors state, “The majority of ants walk e) straighter and f) faster on lower footprint concentrations…”, but this implies that more than 50% of tracks were straighter and or faster on lower "footprint concentrations". The results seem to show that more than 50% showed no significant correlation between straightness and "footprint concentration" and more than 78% showed no significant correlation between speed and "footprint concentration." Please reword this to more accurately reflect the observed pattern.
Fixed.

Figure S3.5: Shift panels c-f down so that the heatmap legend values don't overlap with the two panels above.
Fixed.

Figure S3.12a: This figure panel is missing the numerical values and tick-marks on the x-axis.
Fixed.

Table S4.4 caption: Do you mean “faster” (instead of slower)? Also, note that this statement is based on a statistically insignificant result.
Fixed, reporting no significant effects.

Table S4.10 caption: This claim is only true for the results of the first bin. Consider rewording to reflect the findings more accurately.
Fixed, after rerunning the analysis.

Table S4.11 caption: This caption should refer to speed vs. footprints and note that the relationship is more complicated.
Fixed, after rerunning the analysis.

Figure S5.1: The figure label/number should be S5.3 (according to references in Tables S5.1 & S5.2 above). Also, as in Figure S3.3, the authors state, “The majority of ants walk e) straighter and f) faster on lower footprint concentrations…”, but, again, this implies that more than 50% of tracks were straighter and or faster on lower "footprint concentrations". The results seem to show that 50% showed no significant correlation between straightness and "footprint concentration" and more than 58% showed no significant correlation between speed and "footprint concentration." Please reword this to reflect the observed pattern more accurately.
Fixed.

Table S5.4 caption: This claim is not statistically significant according to the results. Consider rewording to reflect the findings more accurately.
Fixed.

Figure S5.5: Shift panels c-f down so that the heatmap legend values don't overlap with the two panels above.
Fixed.
