# Supplementary material for: Collective search in ants: Movement determines footprints, and footprints influence movement
Source: PLoS One. 2024 Apr 23;19(4):e0299432. doi: 10.1371/journal.pone.0299432 (PMC11037541; doi:10.1371/journal.pone.0299432)
Supplement: Attachment — Submitted filename: Reply to reviews #2.pdf [file pone.0299432.s007.pdf]

Our responses are in blue.

Reviewer #1: I just read the second version of the manuscript now entitled “Searching ants do not avoid chemical footprints, but geometric artifacts have large effects and make causal effects of pheromone hard to detect”.

This version seems to me better than the previous one. The authors answered all my concerns successfully and, thus, I do not have major comments. My only observation is that I still found the title a little confusing. I recommend rewriting it to emphasize the take-home message of the work simply and more directly. Maybe a version of the first subtitle of the discussion section such as “Movement determines footprints, and footprints influence movement: collective searching in the ant…” (or something like that) may work

Thank you for your positive evaluation and the suggestion for a great title, which we adapted slightly modified.

Reviewer #3: In this work, St. Popp and Dornhaus examine the role of previous ant presence on ant movement decisions. Specifically, they reasonably assume that ants passively deposit cuticular hydrocarbons (CHC) as they walk, and after allowing Temnothorax ants to walk in a large arena for 5 hours, correlated speed and path straightness with (presumed) CHC concentrations. They report that ants walk (slightly) faster and straighter on lower CHC levels. They highlight several non-biological explanations for this, but report that some effect remains even when controlling for the non-biological effects.

This is the first time I am reviewing this manuscript. I note that this is a resubmission, but have not looked at the previous reviewer comments or responses, to remain unbiased by them. I am not qualified to review the technical aspects of this work (tracking with Trex, data processing). As this manuscript was submitted to PLoS One, I will refrain from commenting on the subjective interest level of this work.

The work asks reasonable questions, re-examining a topic others have looked at before, but with an unusually rich dataset, and with a more critical eye to non-biological explanations. The writing is generally clear. Some of the methods require a bit more explanation (See detailed comments). The conclusions broadly follow from the results.

We are grateful for your helpful and insightful comments and believe that addressing these has increased the clarity of the manuscript.

I have two major concerns – one technical, and one conceptual.
The technical concern concerns the procedure used to ‘control’ for innate attractiveness of a patch, and thus for the non-biological explanations. The authors “assumed the first visit to each

2x2 mm pixel to be reflective of said properties” (line 318). I don't think this is a robust enough assumption to hang a predictive claim on. I'm not saying there is a better option - I can't think of one. But even if there is a correlation between the first pixel visit and some property of the pixel, it will be so noisy as to be almost not there - purely descriptive. I don’t think it’s reasonable to write unqualified claims about this in the abstract of the manuscript.

We agree with the sentiment and do no longer mention the second effect in the abstract.

My second, conceptual, issue, regards effect sizes. These are indeed reported, but not on any useful, easily-comprehensible scale. I strongly advise providing comprehensible effect size descriptions, such as “the top 10% most visited pixels had XXX% slower movement speeds and XXX% lower sinuosity than the average pixel” or something similar. I will provide some further examples in the detailed comments. Looking at the figures, it seems that the effect sizes are uniformly very small. This (if it is the case) needs to be highlighted in the text, especially the abstract and discussion, by the addition of adjectives such as “slightly”, “small”, and “minor”. This strongly changes what readers will take away from the manuscript. In the conclusions, the authors make some very important statements, which I think are otherwise hidden: that various non-interactive effects (such as environmental idiosyncrasies) are likely to have an overwhelmingly larger effect of movement patterns than any putative ‘home range marking pheromone’ effect. I think this experiment shows that very nicely! But this barely comes out in the abstract. Rather, the abstract focuses on, to me, possibly biologically unimportant effects, while ignoring the more interesting message. All if this is hard to be certain of without some clearer description of the effect sizes.

We added the suggested qualifying words to the abstract to convey the effect size information, and replaced the mentioning of the “second” effect with that of the high sensitivity to environmental features.

I also found the discussion, and indeed the whole paper, a bit on the long and rambling side. However, this is a personal taste thing.
Overall, the study is well designed and well suited to filling a gap in the literature. I have no doubt that it should be published, but am a bit concerned that the results do not strongly support all the conclusions drawn.

MINOR COMMENTS
Title – “geometric artifacts” is not intuitive.
We changed the title to “Collective search in ants: Movement determines footprints, and footprints influence movement”, following from a suggestion of Reviewer #1

Line 81 – I think a lot of readers might be confused when calling these CHCs pheromones. It’s a

reasonable term, but readers are used to thinking of trail or alarm pheromones in ants. Perhaps be explicit first that you are terming them 'pheromones'. Also somewhere it is important to explicitly mention that this species does not use a recruitment pheromone.

We clarified the terminology and added a sentence on the lack of mass recruitment pheromone use in the Study species section.

Line 127 5-20cm is a big range! Why such a range?

Good point: 15 cm away from one of the walls there was a strip of adhesive tape on the underside of the uppermost paper layer, apparently acting as a 'chemical wall' to most ants, inducing thigmotaxis. We thus changed this sentence to: "Points less than ca. 5 cm from the walls or an apparently repellent tape strip on the underside of the top paper layer were excluded,..."

Lines 130-132 – This was not easy for me to understand. Moreover, it was not clear why this was done. This seems like a major alteration to the data, so the motivation should be clear.

"To avoid spurious angles resulting from tracking imprecision of still or stopping ants without using arbitrary thresholding,..." was added to that sentence, as well as just following: Resampling high-frequency movement tracks is an important step for analyzing the data on the biologically most meaningful scale [Tourtellot et al. 1991, J Theor. Biol.]".

Line 153 – reanalysis with 5 minutes and 5 hours – excellent! A really robust approach. I applaud this.

Thank you!

Line 167 – was 'ant' included as a random effect, since ants provide multiple paths?

No, since we do not know which trajectory fragments belong to which ant.

Line 170 – Matlab, eh? Can this produce an analysis document? Like a knitted Rmarkdown file? Would be good in the spirit of open science. I note that as the data was also provided as part of the matlab file, I cannot access it or examine it, since I do not own Matlab. R is free and open source, you know?

We regret that our choice of the analysis software hinders open science and will use better alternatives in the future. The data are provided as .txt and we now also uploaded a markdown file of the MATLAB script to the OSF folder.

Table 1 and elsewhere – "st~FP" is meaningless. Please use intelligible terms. I assume FP is footprints? St is straightness?. Same for lines 250 and elsewhere

Correct, we straightened this out.

Line 201 – only a monster would separate the figure legends from the figures, and have the figures hidden in the back. You're not a monster, are you? I can only assume PLoS One insisted on this. They, then, must be the monsters. No, but seriously, please next time keep the figures with the legends in the main text.

WE are indeed not the monsters here and would like to second the appeal to PLoS to change their guidance on this issue.

Lines 212-217 – this is an excellent paragraph; useful and clear. Can you please provide these sort of summary statistics for the collective effects?

We added the % increase.

Line 251 – here for example adding something like "ants moved XXX% faster away from the nest". But throughout please illustrate the effect sizes.

We added easily interpretable numbers where possible.

Lines 294 – is this because most pixels only ever get walked over once? Maybe we are seeing "slower more torturous ants spend longer at a location"?

There will only be one footprint counted per ant and pixel crossing, such that this correlation is unlikely to explain that "...the less straight and the slower the first ant walked over that pixel, the more footprints accumulated in the following time".
As a response to your word choice, we must note that no ants in our experiments showed abusive behavior towards other ants or the experimenters.

Line 311 – delete 'to'

Done.

Line 392 – some sort of typo

Indeed, we forgot to finish this s. We completed it to say "We analyzed the data from the 'cleaned' trials as if there were still footprints present, to see if the pheromones are the cause of the behavior changes."

Lines 405 "…ants walk a tiny bit straighter and faster…" etc

Done.

Lines 496-500 – I agree completely, and feel like this should be a bigger message of the paper.

We hope that this is satisfactorily addressed by mentioning it in the abstract.

Lines 518 and thereafter – some citation issues going on here (2020)(5).

We included the year of the study in the text to distinguish it from the Hunt et al. 2016 study, while the numbers in brackets are the ‘actual’ citation in the journal’s style.

Line 575 - small b

done.
